# Atomically Engineered Defect‐Rich Palladium Metallene for High‐Performance Alkaline Oxygen Reduction Electrocatalysis

**DOI:** 10.1002/advs.202405187

**Published:** 2024-08-19

**Authors:** Yupeng Zhao, Zhengfan Chen, Nana Ma, Weiyi Cheng, Dong Zhang, Kecheng Cao, Fan Feng, Dandan Gao, Rongji Liu, Shujun Li, Carsten Streb

**Affiliations:** ^1^ Department of Chemistry Johannes Gutenberg University Mainz Duesbergweg 10–14 55128 Mainz Germany; ^2^ Institute of Inorganic Chemistry I Ulm University Albert‐Einstein‐Allee 11 89081 Ulm Germany; ^3^ Henan Key Laboratory of Boron Chemistry and Advanced Materials School of Chemistry and Chemical Engineering Henan Normal University Xinxiang 453007 China; ^4^ School of Physical Science and Technology ShanghaiTech University Shanghai 201210 China; ^5^ Helmholtz‐Institute Ulm Electrochemical Energy Conversion Helmholtzstr. 11 89081 Ulm Germany

**Keywords:** defect engineering, electrocatalysis, metallene, oxygen reduction reaction, zn‐air battery

## Abstract

Defect engineering is a key chemical tool to modulate the electronic structure and reactivity of nanostructured catalysts. Here, it is reported how targeted introduction of defect sites in a 2D palladium metallene nanostructure results in a highly active catalyst for the alkaline oxygen reduction reaction (ORR). A defect‐rich WO_x_ and MoO_x_ modified Pd metallene (denoted: D‐Pd M) is synthesized by a facile and scalable approach. Detailed structural analyses reveal the presence of three distinct atomic‐level defects, that are pores, concave surfaces, and surface‐anchored individual WO_x_ and MoO_x_ sites. Mechanistic studies reveal that these defects result in excellent catalytic ORR activity (half‐wave potential 0.93 V vs. RHE, mass activity 1.3 A mgPd^−1^ at 0.9 V vs. RHE), outperforming the commercial references Pt/C and Pd/C by factors of ≈7 and ≈4, respectively. The practical usage of the compound is demonstrated by integration into a custom‐built Zn‐air battery. At low D‐Pd M loading (26 µgPd cm^−2^), the system achieves high specific capacity (809 mAh g_Zn_^−1^) and shows excellent discharge potential stability. This study therefore provides a blueprint for the molecular design of defect sites in 2D metallene nanostructures for advanced energy technology applications.

## Graphical abstract

Three unique atomic‐scale defects, that are pores, concave surfaces, and surface‐anchored WO_x_ and MoO_x_ sites, are incorporated into an ultra‐thin Pd metallene. This structure demonstrates outstanding oxygen reduction reaction performance and enhanced stability.

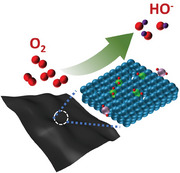

## 1 Introduction

The oxygen reduction reaction (ORR) is one of the most important catalytic processes for energy conversion and storage technologies including fuel cells, metal‐air batteries, and electrochemical H_2_O_2_ production.^[^ ^1^, ^2^, ^3^ ^]^ The ORR is a challenging reaction, due to the high O_2_ bond dissociation energy (498 KJ mol^−1^) and the sluggish kinetics of O_2_‐related proton‐coupled multi‐electron processes.^[^ ^2^, ^4^ ^]^ These factors significantly impede the practical application of the ORR. To overcome these challenges, numerous electrocatalysts have been developed, including those based on platinum group metals (PGMs),^[^ ^5^ ^]^ noble metal‐free materials,^[^ ^6^, ^7^ ^]^ and even metal‐free catalysts.^[^ ^8^ ^]^ Currently, both the intrinsic activity and the long‐term stability of noble metal‐free and metal‐free catalysts are significantly inferior to those of PGM‐based electrocatalysts.^[^ ^4^ ^]^

Owing to their unique electronic structure and optimum oxygen molecule adsorption energy, electrocatalysts based on PGMs have received widespread attention and are still a focal point for advanced ORR catalyst development.^[^ ^9^ ^]^ However, challenges remain, particularly in the case of platinum‐based catalysts. While these materials demonstrate excellent catalytic performance, they often suffer from poor long‐term stability.^[^ ^10^, ^11^, ^12^ ^]^ Importantly, highly dispersed Pt nanoparticles show a notable tendency to aggregate during catalysis, resulting in the formation of larger particles with reduced reactivity.^[^ ^13^ ^]^ Furthermore, the high binding energy of Pt and Pd to oxygen molecules limits the generation and conversion of intermediates (such as *OH and *OOH) into H_2_O/OH^‐^, indicating potential opportunities for enhancing the catalytic properties by modification in their electronic structure.^[^ ^14^, ^15^ ^]^ Moreover, the substantial cost associated with Pt and Pd is major impediment to their broad industrial use.^[^ ^16^ ^]^ Thus, chemical strategies are urgently required which optimize electronic structure and reactivity of PGMs while at the same time making optimum use of all metal centers available in the catalyst.^[^ ^17^, ^18^, ^19^ ^]^

These challenges have recently been targeted by the development of two‐dimensional (2D) metal nanosheets, so‐called metallenes, which have shown unique electronic structure and high reactivity and durability.^[^ ^20^, ^21^, ^22^ ^]^ Metallenes are metal or alloy materials featuring individual layers of atomic thickness.^[^ ^23^, ^24^ ^]^ Metallenes offer optimum utilization of the individual metal atoms due to their high specific accessible surface area and abundance of low‐coordinated reactive metal sites.^[^ ^25^, ^26^, ^27^ ^]^ A significant breakthrough in this realm was achieved by Guo and colleagues, who successfully synthesized PdMo bimetallene.^[^ ^28^ ^]^ The group discovered that strain and quantum effects within the ultrathin structure of the metallene lead to a downward shift of the d‐band center, thereby lowering the oxygen molecule binding energy and enhancing electrocatalytic performance. Moreover, the electronic structure can be further modulated by doping with Mo atoms. As a result, PdMo bimetallene exhibits a lower d‐band center and superior ORR catalytic activity compared to pure Pd metallene. Building on this advancement, Jin and co‐workers further demonstrated that the durability of PdMo bimetallene can be significantly improved by interstitial doping with carbon. This modification results in stabilization of the Mo sites within the bimetallene structure.^[^ ^29^ ^]^ In addition, defect engineering has proven to be a critical tool in the advancement of Pd metallene. Wang and colleagues successfully developed a defect rich Pd metallene characterized by abundant pores, which created a multitude of highly active sites. This innovation resulted in a significant enhancement in ORR activity.^[^ ^30^ ^]^ Concurrently, research by Guo and coworkers revealed that the concave surfaces on Pd metallene lead to a modest downward shift in the d‐band center and allow fine‐tuning of oxygen molecule binding energies. This structural modification contributes to improved ORR performance, showcasing the impact of the surface geometry on catalytic efficiency.^[^ ^31^ ^]^

Here, we build on these pioneering studies and report a novel ultrathin Pd metallene doped with atomic WO_x_/MoO_x_ (referred to as **D‐Pd M**) featuring by a curved defect‐rich structure. Key for the synthetic access to this new compound is the use of molecular metal oxides (polyoxometalates, POMs), here reduced [H_3_PMo_12_O_40_] ∙ x H_2_O ( = PMo_12_), N, N‐dimethylformamide (DMF), and tungsten hexacarbonyl (W(CO)_6_) as reducing agents. Meawhile, W(CO)_6_ was used as a structure‐directing agent. PMo_12_ and W(CO)_6_ also acted as molecular precursors to deposit MoO_x_ and WO_x_ into the Pd metallene. The resulting **D‐Pd M** showed excellent ORR activity, with a high half‐wave potential (*E*_1/2_ = 0.93 V vs. RHE), high mass activity (1.3 A mg_Pd_^ −1^ at 0.9 V vs. RHE) and superior stability over 10,000 test cycles. Practical applicability of **D‐Pd M** was demonstrated by integration into an operational Zn‐air battery at ultra‐low Pd loading (26 µg_Pd_ cm^−2^), showing high specific capacity (809 mAh g_Zn_^−1^), exceptional discharge performance and ultra‐long cycling stability (continuous operation for 300 h over 300 charge/discharge cycles).

## 2 Results and Discussion

**Figure 1:**
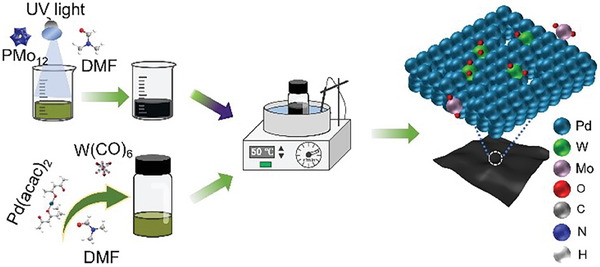
Schematic illustration for the fabrication of **D‐Pd M**.

**Figure** **1** illustrates the synthetic procedure to access **D‐Pd M**. Initially, a solution of PMo_12_ in DMF was irradiated with ultraviolet (UV) light (l_max_ = 254 nm) to reduce the POM cluster. The solution undergoes a clear transition from its initial yellow to dark blue color, and a new broad peak at 760 nm appeared in the reduced solution (Figure S1, Supporting Information), all of which suggests that the POM cluster was reduced to heteropoly blue.^[^ ^32^, ^33^ ^]^ The solution containing the reduced PMo_12_ was then combined with a DMF solution containing the metal precursors palladium (II) acetylacetonate (Pd(acac)_2_) and W(CO)_6_, resulting in reduction of Pd^2+^ to Pd (0) and formation of the palladene sheets.^[^ ^30^ ^]^ During the reaction, carbon monoxide was gradually released by thermal decomposition of W(CO)_6_. CO is known to strongly adsorb on Pd (111) facet, which in turn facilitates the anisotropic lateral growth and formation of 2D ultrathin metallene.^[^ ^23^, ^31^, ^34^ ^]^ For comparison, conventional Pd metallene (**Pd M**) weas prepared using a modified literature method, see Supporting Information (SI),^[^ ^34^ ^]^ where citric acid was used as the reducing agent instead of PMo_12_.

**Figure 2:**
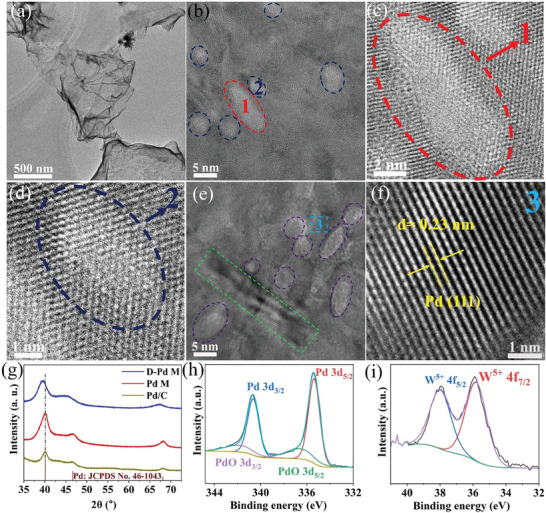
a) TEM image and b,e) HRTEM images of **D‐Pd M**, with corresponding enlarged crystal details shown in c,d,f) for selected areas (area 1, area 2, and area 3, respectively). g) XRD patterns of **D‐Pd M**, Pd M, and Pd/C. h) Deconvoluted XPS spectra of Pd 3d and i) W 4f of **D‐Pd M**.

The morphology and structural properties of the as‐synthesized **D‐Pd M** and Pd M were characterized by transmission electron microscopy (TEM) and high‐resolution transmission electron microscopy (HRTEM). As shown in **Figure** **2**a, D**‐Pd M** exhibits an ultrathin 2D nanosheet structure with a lateral diameter of ≈1 µm. In contrast, Pd M exhibits a hexagonal nanosheet structure with an average diameter of 70 nm (Figure S2, Supporting Information). Selected area electron diffraction (Figure S3, Supporting Information) confirms the presence of a face‐centered cubic (fcc) structure, and atomic force microscopy measurements (Figure S4, Supporting Information) give an average thickness of ≈0.9 nm, suggesting the presence of 3 to 5 Pd atom layers.^[^ ^28^ ^]^ TEM energy‐dispersive X‐ray spectroscopy (TEM‐EDX; Figure S5, Supporting Information) shows the homogenous distribution of Pd, W, and Mo throughout the metallene. The atomic ratio of Pd:W:Mo was determined by inductively coupled plasma optical emission spectroscopy (ICP‐OES) to be 93.8: 5.2: 1. Detailed morphological analysis of the TEM data reveals curved surface with abundant defects, including pores and concave structures (Figure S6, Supporting Information), which could be due to the oxidative etching of O_2_ and PMo_12_ in the highly acidic environment provided by PMo_12_.^[^ ^26^, ^31^ ^]^ The defects were further probed by HR‐STEM. Figure 2b shows the presence of pores (red circle, **area 1**) and concave areas (dark blue circle, **area 2**), also see Figure S7 (Supporting Information).^[^ ^15^ ^]^ The brighter contrast observed in Figure 2b is indicative of the lower thickness in these areas compared to their surroundings. Notably, in the higher magnification of **area 1** (Figure 2c), step atoms at the inner edge can be observed. Meanwhile, in Figure 2d, the clear atom distribution and brighter contrast indicate the presence of a concave structure.^[^ ^15^ ^]^ Numerous concave structures with various diameters, typically less than 10 nm, are observed in Figure 2e (dark blue circles). We also use a false‐color mode to illustrate the thickness contrast more clearly (Figure S8a–d, Supporting Information). Additionally, curved structures are also observed in HRTEM (green rectangles in Figure 2e; Figure S8e, Supporting Information), which leads to lattice distortion. The fast Fourier transformation (FFT) pattern (Figure S8f, Supporting Information) exhibits a sixfold symmetric fcc structure, implying that the metallene is stacked dominantly along the (111) facets.^[^ ^15^ ^]^ TEM analyses give a lattice spacing of 0.23 nm (Figure 2f), which exceeds that of bulk Pd, suggesting the presence of intrinsic tensile strain due to its atomically thin structure.

The results are consistent with powder X‐ray diffraction (pXRD) analyses shown in Figure 2g, where the peak at 39.4° is attributed to the (111) plane of the fcc structure of Pd metallene. Additionally, smaller peaks corresponding to the (200) plane at 46.7° and the (220) plane at 68.1° in **D‐Pd M** compared to Pd M and Pd/C indicate the predominance of the (111)‐exposed feature. Furthermore, the shifts to lower 2θ angles observed in **D‐Pd M** compared to Pd M and Pd/C are attributed to strain effects and lattice distortions caused by the defect structure. Next, the oxidation states of **D‐Pd M** were investigated by X‐ray photoelectron spectroscopy (XPS). The deconvoluted spectrum of Pd 3d shows the distinct characteristic peaks centered at 335.4 and 340.7 eV, which are assigned to Pd (0) 3d_5/2_ and Pd (0) 3d_3/2_, respectively (Figure 2h). The small peaks at 336.7 and 342 eV are assigned to PdO, assigned to surface oxidation of Pd.^[^ ^25^, ^26^ ^]^ The results indicate that **D‐Pd M** is dominated by metallic Pd. The deconvoluted W 4f spectrum (Figure 2i) shows the existence of W^5+^ species.^[^ ^35^ ^]^ In contrast, the oxidation state of Mo cannot be assigned reliably by XPS due the low concentration of Mo species in **D‐Pd M**.

**Figure 3:**
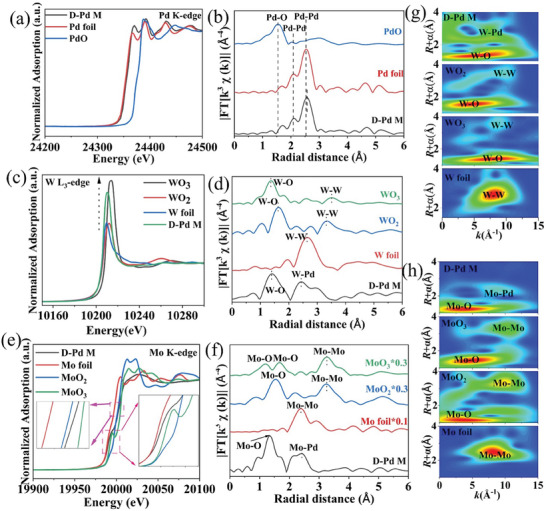
XAS characterizations of **D‐Pd M**. Experimental XANES spectra a,c,e) and EXAFS spectra b,d,f) of Pd, W, and Mo, respectively. Wavelet transform profiles of W g) and Mo h).

X‐ray absorption spectroscopy (XAS) characterization was performed to further investigate the chemical states and local coordination environments of **D‐Pd M**. The normalized Pd K‐edge X‐ray absorption near‐edge structure (XANES) spectrum (**Figure** **3**a) of **D‐Pd M** is similar to the Pd foil reference, indicating its dominant metallic state,^[^ ^25^, ^26^ ^]^ which coincides with the XPS result. Fourier transform extended X‐ray absorption fine structures (FT‐EXAFS) analysis was employed to investigate the local environment of the Pd atoms (Figure 3b). The strong peak in the **D‐Pd M** EXAFS spectrum at 2.57 Å is assigned to the Pd‐Pd pairs.^[^ ^28^ ^]^ The average coordination number (CN) of elements is proportional to the intensity of FT‐χ(k) peak and the corresponding fitting results of Pd K‐edge FT‐EXAFS (Figure S9 and Table S1, Supporting Information) give an average CN of 10.7 ± 1.0, which is lower than that in Pd foil (CN_Pd foil_ = 12), indicating the existence of unsaturated Pd sites. This is in agreement with the proposed thin‐layered structure and the presence of multiple defects in the **D‐Pd M**.^[^ ^31^ ^]^ The W species in **D‐Pd M** was further investigated by analyzing its L_3_‐edge XANES and FT‐EXAFS spectra. As shown in Figure 3c, the white‐line peak intensity is between WO_2_ and WO_3_, which gives an approximate oxidation state of +5.^[^ ^35^, ^36^ ^]^ This finding is consistent with the results obtained from XPS analysis. In addition, the peaks in the FT‐EXAFS spectrum (Figure 3d) at 1.38 and 2.43 Å can be assigned to W‐O and W‐Pd environments.^[^ ^36^, ^37^ ^]^ The corresponding fitting results of W L_3_‐edge FT‐EXAFS show the CN of W‐Pd is 6.3 ± 0.8. Based on these results, we propose that WO_x_ (x = 2.5) is doped on the surface of metallene. Additionally, W substitutes the position of Pd, as illustrated in Figure S12 (Supporting Information).^[^ ^38^ ^]^ Notably, while W‐W pairs are present in bulk WO_2_ and WO_3_, they are not observed in **D‐Pd M** according to the FT‐EXAFS spectrum and wavelet transform analysis (Figure 3g), which can better resolve features in K‐space and radial distance. The results indicate that atomic WO_x_ species are doped on the surface of the metallene. Furthermore, we also investigated the chemical states and local coordination environments of Mo in **D‐Pd M** by employing MoO_2_, MoO_3_, and Mo foil as reference materials. In Figure 3e, MoO_3_ exhibits a distinct pre‐edge feature at ≈19,995 eV, which is not observed in MoO_2_ and Mo foil. Additionally, a weak pre‐edge is observed in **D‐Pd M**, suggesting that the oxidation state of Mo in **D‐Pd M** falls between that of MoO_2_ and MoO_3_.^[^ ^39^ ^]^ Furthermore, the near‐edge structure XANES spectra also support this conclusion. The FT‐EXAFS spectrum in Figure 3f indicates the presence of Mo‐O and Mo‐Pd pairs, with no Mo‐Mo species observed (Figure 3h), suggesting atomic dispersion of MoO_x_. The fitting results of Mo K‐edge FT‐EXAFS for Mo‐Pd give a CN of 0.9 ± 0.2, indicating that the MoO_x_ are located on the surface of the Pd sheets rather than being embedded in the Pd lattice, as illustrated in Figure S12 (Supporting Information).^[^ ^39^ ^]^ More detailed peak assignments for FT‐EXAFS of Pd, W, and Mo can be found in Table S1 (Supporting Information). The comparison of corresponding fitting results and the raw data can be found in Figures S9–S11 (Supporting Information).

**Figure 4:**
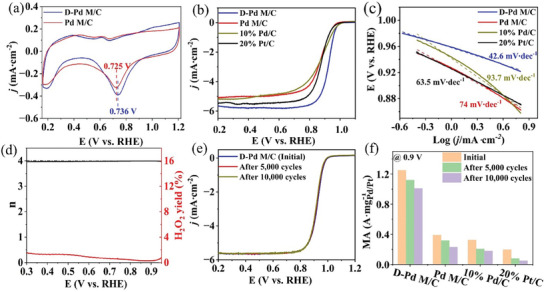
a) CVs of **D‐Pd M**/C and Pd M/C, b) LSVs, and c) Tafel slopes of **D‐Pd M**/C, Pd M/C, Pd/C, and Pt/C, d) electron transfer number and H_2_O_2_ yield of **D‐Pd M**, e) LSVs of **D‐Pd M**, and f) mass activities of **D‐Pd M**/C, Pd M/C, Pd/C, and Pt/C at 0.9 V before and after 5,000 and 10,000 cycles.

In the next step, we evaluated the electrocatalytic performance of the **D‐Pd M** for ORR in 0.1 m aqueous KOH using a typical three‐electrode electrochemical setup. Here, the saturated calomel electrode was used as the reference electrode, and a graphite rod was employed as the counter electrode. All potentials in this study have been converted to reversible hydrogen electrode potentials (RHE, see details in the Supporting Information). Prior to the measurement, freshly prepared **D‐Pd M** and Pd M were deposited on a VULCAN® XC‐72 carbon support (See details in Supporting Information). In addition, commercial 10% Pd/C and 20% Pt/C were also used as ORR catalyst references. The respective catalysts ink was drop‐cast onto the glassy carbon rotating disk electrode, which acted as the working electrode. The metal loading on the electrode for **D‐Pd M**/C and Pd M/C was controlled to be 10 µg cm^−2^, while that of commercial Pd/C and Pt/C was controlled to be 15 µg cm^−2^ to reach the well‐defined limiting current densities. The typical cyclic voltammetry (CV) curves of all the catalysts were recorded in N_2_‐saturated 0.1 m KOH at a scan rate of 50 mV s^−1^. As shown in **Figure** **4**a and Figure S13 (Supporting Information), the peak located at 0.74 V of **D‐Pd M**/C is assigned to Pd oxide reduction.^[^ ^28^ ^]^ Notably, the reduction potential is a descriptor of the Pd‐O binding energy. Compared to Pd/C (0.69 V) and Pd M/C (0.73 V), the peak of **D‐Pd M**/C shifts positively, revealing its weaker oxygen affinity.^[^ ^27^, ^40^ ^]^ Next, we determined the electrochemically active surface areas (ECSAs) of **D‐Pd M**/C and reference catalysts by underpotentially deposited hydrogen (H_upd_) and underpotentially depositing copper (Cu_upd_) methods (See method details in Supporting Information). The ECSAs determined from the Cu_upd_ method were used for further analysis. The ECSA values are estimated by Cu_upd_ to be 79 m^2^ g_Pd_^−1^ for **D‐Pd M**/C, 70.5 m^2^ g_Pd_^−1^ for Pd M/C, 40.4 m^2^ g_Pd_^−1^ for Pd/C, and 51.8 m^2^ g_Pt_^−1^ for Pt/C, respectively (Figure S14, Supporting Information). The increased active areas of **D‐Pd M** can be attributed to its porous and curved structure. This structural characteristic ensures that more active sites are exposed to the electrolyte during the catalytic process.

The linear sweep voltammetry (LSV) curves of all the samples were determined in an O_2_‐saturated 0.1 m KOH electrolyte at a scan rate of 20 mV s^−1^ to assess their ORR performance. As shown in Figure 4b, D**‐Pd M**/C shows the most positive half‐wave potential (E_1/2_, 0.93 V) and onset potential (E_onset_, 1.02 V), as compared with those of Pd M/C (0.87 and 0.99 V), Pd/C (0.87 and 1.02 V), and Pt/C (0.88 and 1.01 V), revealing that **D‐Pd M**/C exhibited the best catalytic performance for ORR among all the catalysts. The diffusion‐limiting current density (***j*_L_**) of **D‐Pd M**/C (5.7 mA cm^−2^) is also higher than that of Pd M/C (5.4 mA cm^−2^), Pd/C (5.5 mA cm^−2^), and Pt/C (5.6 mA cm^−2^). Such high limiting current density is a benefit to the high‐power densities of Zn‐air batteries. Besides, **D‐Pd M**/C has the smallest Tafel slope value (Figure 4c) of 42.6 mV dec^−1^, compared with that of Pd M/C (74 mV dec^−1^), Pd/C (93.7 mV dec^−1^), and Pt/C (63.5 mV dec^−1^), which indicates faster ORR reaction kinetics on the surface of **D‐Pd M**. To further quantify the intrinsic ORR activity, kinetic currents were calculated for all catalysts using the Koutecký‐Levich (K‐L) equation (see details in Supporting Information). These were then normalized based on the metal mass loading of the catalysts and the ECSA to determine the mass avtivity (MA) and specific activity (SA). As shown in Figure S15 (Supporting Information), **D‐Pd M**/C achieves an MA of 1.3 A mg_Pd_^−1^ at the generally chosen potential of 0.9 V, which is 3.3, 3.9, and 6.5 times higher than that of Pd M/C, commercial Pd/C, and commercial Pt/C. Additionally, **D‐Pd M**/C delivers an MA of 5.2 A mg_Pd_^−1^ at 0.85 V. This value is also apparently higher than that of Pd M/C (1.4 A mg_Pd_^−1^), commercial Pd/C (0.88 A mg_Pd_^−1^), and commercial Pt/C (0.88 A mg_Pt_^−1^). Likewise, **D‐Pd M**/C also has specific activities of 6.13 and 1.5 mA cm^−2^ at 0.85 and 0.9 V, respectively, which significantly outperform Pd M/C (1.86 and 0.52 mA cm^−2^), commercial Pd/C (2.46 and 0.91 mA cm^−2^), and commercial Pt/C (1.31 and 0.3 mA cm^−2^). The excellent ORR catalytic performance of **D‐Pd M**/C can be attributed to the lower Pd‐O binding energy, which facilitates the generation of intermediates (*OH and *OOH) and their transformation to H_2_O. Furthermore, to understand the catalytic process of **D‐Pd M**, rotating ring‐disk electrode measurements were used to determine the electron transfer number (n) and hydrogen peroxide yield, see details in Supporting Information. As shown in Figure 4d and Figure S16a (Supporting Information), the electron transfer number is >3.9 between 0.3 to 0.95 V, revealing a four‐electron transfer‐dominated pathway, which agrees well with the results obtained from the K‐L plot at rotation rates between 625 and 2025 rpm (Figure S16b,c, Supporting Information). The H_2_O_2_ yield is only 0–1.6% within the potential range from 0.8 to 0.3 V. Pd M/C, Pd/C, and Pt/C exhibit similar results, indicating a consistent four‐electron transfer process (Figure S17, Supporting Information).

Apart from electrocatalytic activity, stability is another essential criterion for the comprehensive evaluation of the catalytic performance. Therefore, we employed accelerated durability tests by CV cycling between 0.6 and 1.0 V at a scan rate of 200 mV s^−1^ in an O_2_‐saturated 0.1 m KOH electrolyte. As shown in Figure 4e, after 5,000 and 10,000 cycles, the E_1/2_ of **D‐Pd M**/C shows only negligible decay (4 and 6 mV, respectively). In contrast, an apparent negative shift was observed for Pd M/C (10 and 23 mV), commercial Pd/C (19 and 23 mV), and commercial Pt/C (25 mV and 38 m), respectively (Figure S18, Supporting Information). In addition, the MA of **D‐Pd M** /C at 0.9 V has decreased by 10.4% and 19.2% after 5,000 and 10,000 cycles (Figure 3f), which outperforms those of Pd M/C (18.7% and 41%), commercial Pd/C (36.6% and 44.6%), and commercial Pt/C (60.4% and 74.6%). The same scenario was also observed at 0.85 V vs. RHE in all the catalysts (Figure S19, Supporting Information). Additionally, the morphology and structure of **D‐Pd M** have no obvious degradation after 10,000 cycles, compared to the pristine sample. In comparison, the apparent aggregation of Pt nanoparticles was observed in Pt/C (Figure S20, Supporting Information). The composition of D‐Pd M was determined by ICP‐OES, which shows the atomic ratio of Pd:W is 94.2:5.8. However, no Mo was determined, which indicates that MoO_x_ dissolved under the electrochemical tests and explained the slight catalytic degradation.

**Figure 5:**
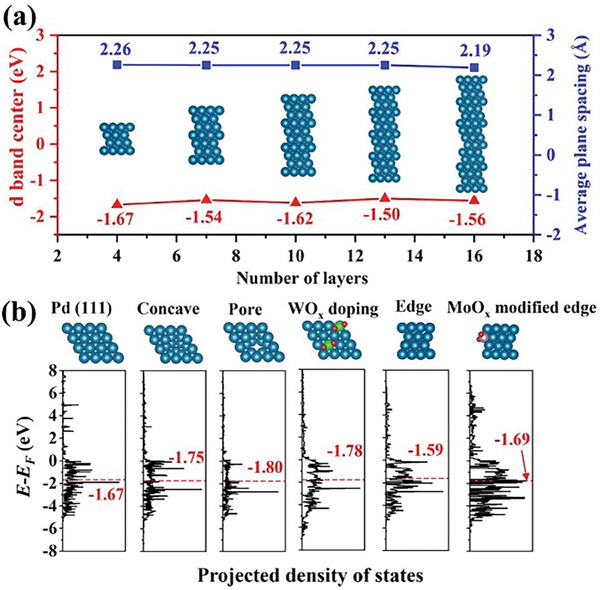
DFT calculations of *d*‐band centers. a) The *d*‐band center of surface atoms and average plane spacings of Pd nanosheets with 4, 7, 10, 13, and 16 layers. The side view of atomic models is inserted. b) From left to right, the d band centers of surface atoms in Pd (111), exposed sublayer atoms in concave defects, exposed sublayer atoms in pores defects, surface atoms in WO_x_ doping, edge atoms, and MoO_x_ modified edge atoms. The first four schematic illustrations are from the top view and the last two are from side view of atomic model. The navy, green, pink, and red balls represent Pd, W, Mo, and O, respectively.

Based on the previous report from Norskov, the binding energy between catalysts and O_2_ is linearly correlated with the energy center of the valence *d*‐band density of states (*d*‐band center), making it one of the most successful descriptors of ORR activity.^[^ ^41^ ^]^ We therefore calculated the *d*‐band centers of various defects in **D‐Pd M** using density functional theory (DFT) to elucidate the reasons for the superior ORR activity of **D‐Pd M**. We constructed a four‐atom layer model for the **D‐Pd M**, incorporating WO_x_ doping into the surface lattice of metallene and MoO_x_ attachment to the edge Pd atoms. The loading of W and Mo were set to 5% and 1%, respectively, to be in line with experimental results. As shown in **Figure** **5**a, the *d*‐band centers of surface Pd atoms in different layer Pd sheets were calculated. A downward shift (≈0.11 eV) in the *d*‐band center of surface atoms is observed in the four‐layer Pd sheet compared to the sixteen‐layer Pd sheet, which results in less strongly bound O_2_ on the surface Pd atoms.^[^ ^28^ ^]^ We note that the average spacing between the planes in four‐layer Pd metallene is 3.2% greater than in a 16‐layered nanosheet, indicating a higher level of tensile strain in the four‐layer structure compared to multilayer Pd sheets. The electronic structures tuned by defects were also investigated. As illustrated in Figure 5b, in a perfect four‐layer Pd sheet, the *d*‐band center of the surface atoms in the (111) plane is −1.67 eV. However, when subjected to concave defects and pore defects, the sublayer layer atoms feature substantially lower *d*‐band centers (−1.75 and −1.8 eV, respectively) compared to the surface atoms. This indicates a weaker binding ability to O_2_, thereby facilitating the formation and conversion of intermediates (*OH and *OOH) to H_2_O/OH^‐^.^[^ ^14^, ^15^ ^]^ The findings confirm that concave and pore defects serve as effective means to introduce highly active sites, thereby enhancing the catalytic properties for ORR. Moreover, embedding WO_x_ into the surface Pd lattice also leads to a downshift of the *d*‐band center by 0.11 eV. The average *d*‐band center of edge Pd atoms was calculated to be −1.59 eV, significantly higher than that of surface atoms. This discrepancy reveals the less favorable catalytic features for ORR exhibited by edge atoms. After attaching MoO_x_ to the edge Pd atoms, the *d*‐band center of these atoms shifted downward to −1.69 eV, a value similar to the *d*‐band center of surface atoms in Pd (111), indicating the improved catalytic properties. These computational results suggest that size effects, pore defects, concave defects, WO_x_ doping, and MoO_x_ modification can effectively enhance the ORR activity of **D‐Pd M**. We note that these calculations do not consider the leaching of MoO_x_ species during long‐term electrochemical operation (see above).

**Figure 6:**
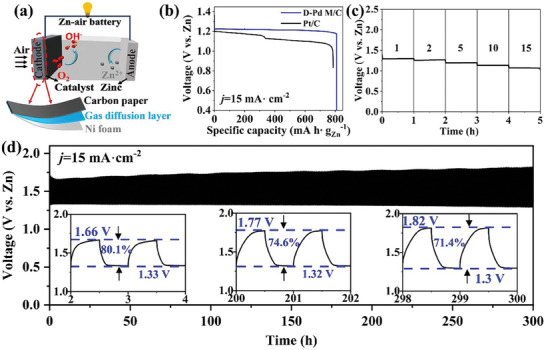
a) Scheme of the aqueous Zn‐air battery, b) full discharge profile (voltage versus specific capacity of discharge) for a Zn‐air battery using **D‐Pd M**/C as the air cathode at a current density of 15 mA cm^−2^, c) the discharge curves of Zn‐air batteries at current densities of 1, 2, 5, 10, and 15 mA cm^−2^, and d) the charge–discharge profiles of two Zn‐air batteries worked at a current density of 15 mA cm^−2^ with each cycle lasting 60 min.

Next, the practical utility of the **D‐Pd M** catalyst was tested by integration into an aqueous custom‐built Zn‐air battery (Figure S21, Supporting Information). Given the economic expense of Pd metal, we opted to test the system at an extremely low Pd loading at the cathode (26 µg_Pd_ cm^−2^). The principal structure of the Zn‐air battery is shown in **Figure** **6**a; briefly, the battery is based on a carbon paper‐supported **D‐Pd M**/C as the air cathode, a Zn plate as the anode, and 6 m aqueous KOH solution containing 0.2 m Zn(CH_3_COO)_2_ as the electrolyte. Initial tests show that the Zn‐air battery achieves a high open‐circuit potential (OCP) of 1.4 V (Figure S21, Supporting Information). The specific capacities of the **D‐Pd M**/C‐ battery at 15 mA cm^−2^ is illustrated in Figure 6b. For comparison, a reference Zn‐air battery using commercial Pt/C coated carbon paper (26 µg_Pt_ cm^−2^) as air cathode was tested under the same conditions. After normalizing for the weight loss of Zn, the **D‐Pd M**/C‐based battery exhibits a discharge specific capacity of 809 mAh g_Zn_^−1^, corresponding to an energy density of 982 Wh kg_Zn_^−1^, which outperforms the Pt/C‐based battery (785 mAh g_Zn_^−1^ and 890 Wh kg_Zn_^−1^). In addition, during the long‐term discharge test, the potential of the **D‐Pd M**/C‐based cell remained remarkably stable and only a slight potential decrease of 0.05 V was observed. In contrast, a notable potential drop (>0.2 V) was observed for the Pt/C‐based battery. The stability of the **D‐Pd M**/C‐based battery is further shown by the discharge curves at various current densities (Figure 6c). Also, the **D‐Pd M**/C‐based battery achieves a high power density (55 mW cm^−2^) compared with the Pt/C‐based battery (37 mW cm^−2^), see Figure S22a (Supporting Information). The power density of the **D‐Pd M**/C‐based battery can be increased by at higher **D‐Pd M** loadings, see Figure S22b (Supporting Information).

To assess the cycling durability, the **D‐Pd M**/C‐based Zn‐air battery was further tested at a charging and discharging rate of 15 mA cm^−2^ for 60 min per cycle. As illustrated in Figure 6d, **D** **‐Pd M**/C‐based battery exhibits a significantly longer cycling lifetime over 300 cycles (≈300 h) with a small charge/discharge voltage gap. Initially, the charge/discharge voltage efficiency reached 80.1% with a potential gap of 0.33 V. During cycling, the voltage efficiency decreased slowly, while the discharging potential remained exceptionally stable throughout the measurement. The gradual increase in charging potential resulted in a decrease in round‐trip voltage efficiency, possibly attributed to imperfect oxygen evolution activity under conditions of very low palladium loading (Figure S22c, Supporting Information).

## 3 Conclusion

In summary, we demonstrate how molecular‐level tuning of 2D metallene structures becomes possible using bottom‐up materials design routes. The study shows how introduction of various defect sites impacts the oxygen reduction reaction reactivity of a 2D palladene nanostructure catalyst, leading to high reactivity and stability under harsh conditions. Combined experimental and theoretical analyses reveal the correlation between defects, change in electronic structure, and resulting reactivity. Integration of the catalyst into as cathode in a Zn‐air battery demonstrated outstanding performance at very low Pd loading. This study offers valuable insights into the design of efficient ORR catalysts with reduced precious metal consumption, which can open new paths for larger‐scale deployment of non‐earth‐abundant elements.

## 4 Experimental Section

The authors have cited additional references within the Supporting Information.^[^ ^31^, ^33^, ^42^, ^43^, ^44^, ^45^, ^46^ ^]^ A preprint of this manuscript has been deposited at https://doi.org/10.26434/chemrxiv‐2024‐fkr00.

## Conflict of Interest

The authors declare no conflict of interest.

## Supporting information

Supporting Information

## Data Availability

The data that support the findings of this study are openly available in Zenodo.org at https://doi.org/10.5281/zenodo.10984539, reference number 10984539.

## References

[1] S. Chu , A. Majumdar , Nature 2012, 488, 294. 22895334 10.1038/nature11475

[2] A. Han , X. Wang , K. Tang , Z. Zhang , C. Ye , K. Kong , H. Hu , L. Zheng , P. Jiang , C. Zhao , Q. Zhang , D. Wang , Y. Li , Angew. Chem., Int. Ed. 2021, 60, 19262. 10.1002/anie.202105186 34156746

[3] C. Lim , A. R. Fairhurst , B. J. Ransom , D. Haering , V. R. Stamenkovic , ACS Catal. 2023, 13, 14874. 38026811 10.1021/acscatal.3c03321 PMC10660348

[4] Z. Ma , Z. P. Cano , A. Yu , Z. Chen , G. Jiang , X. Fu , L. Yang , T. Wu , Z. Bai , J. Lu , Angew. Chem., Int. Ed. 2020, 59, 18334. 10.1002/anie.202003654 32271975

[5] Z. Lyu , X.‐G. Zhang , Y. Wang , K. Liu , C. Qiu , X. Liao , W. Yang , Z. Xie , S. Xie , Angew. Chem., Int. Ed. 2021, 60, 16093. 10.1002/anie.202104013 33884729

[6] Q. Yang , Y. Jia , F. Wei , L. Zhuang , D. Yang , J. Liu , X. Wang , S. Lin , P. Yuan , X. Yao , Angew. Chem., Int. Ed. 2023, 62, 202315752. 10.1002/anie.202000324 31960551

[7] M. A. de Araújo , A. A. Koverga , A. M. P. Sakita , F. B. Ometto , L. G. da Trindade , E. A. Ticianelli , ChemCatChem 2023, 15, 202201594.

[8] J. Zhang , L. iangti Qu , G. Shi , J. Liu , J. Chen , L. Dai , D. Zhang , J. Liu , L. Dai , L. Qu , G. Shi , J. Chen , Angew. Chem., Int. Ed. 2016, 55, 2230. 10.1002/anie.201510495 26709954

[9] Z. Zhao , C. Chen , Z. Liu , J. Huang , M. Wu , H. Liu , Y. Li , Y. Huang , Adv. Mater. 2019, 31, 1808115. 10.1002/adma.201808115 31183932

[10] K. J. Sawant , Z. Zeng , J. P. Greeley , Angew. Chem., Int. Ed. 2024, 63, 202312747. 10.1002/anie.202312747 38133533

[11] E. Hornberger , V. Mastronardi , R. Brescia , P. P. Pompa , M. Klingenhof , F. Dionigi , M. Moglianetti , P. Strasser , ACS Appl. Energy Mater. 2021, 4, 9542.

[12] T. Đukić , L. Pavko , P. Jovanovič , N. Maselj , M. Gatalo , N. Hodnik , Chem. Commun. 2022, 58, 13832. 10.1039/d2cc05377b PMC9753161 36472187

[13] D. Y. Chung , J. M. Yoo , Y. E. Sung , Adv. Mater. 2018, 30, 1704123. 10.1002/adma.201704123 29359829

[14] S. Huang , S. Lu , S. Gong , Q. Zhang , F. Duan , H. Zhu , H. Gu , W. Dong , M. Du , ACS Nano 2022, 16, 522. 34939416 10.1021/acsnano.1c07574

[15] F. Lin , F. Lv , Q. Zhang , H. Luo , K. Wang , J. Zhou , W. Zhang , W. Zhang , D. Wang , L. Gu , S. Guo , Adv. Mater. 2022, 34, 2202084. 10.1002/adma.202202084 35484940

[16] N. Zhang , Q. Shao , X. Xiao , X. Huang , Adv. Funct. Mater. 2019, 29, 1808161.

[17] L. Bu , N. Zhang , S. Guo , X. Zhang , J. Li , J. Yao , T. Wu , G. Lu , J. Y. Ma , D. Su , X. Huang , Science 2016, 354, 1410. 27980207 10.1126/science.aah6133

[18] X. Wang , Z. Li , Y. Qu , T. Yuan , W. Wang , Y. Wu , Y. Li , Chem 2019, 5, 1486.

[19] Y. Yang , W. Xiao , X. Feng , Y. Xiong , M. Gong , T. Shen , Y. Lu , H. D. Abruña , D. Wang , ACS Nano 2019, 13, 5968. 30998846 10.1021/acsnano.9b01961

[20] L. Zhang , Z. Zhao , X. Fu , S. Zhu , Y. Min , Q. Xu , Q. Li , ACS Appl. Mater. Interfaces 2023, 15, 5198. 36691303 10.1021/acsami.2c19196

[21] Q. Yang , L. Shi , B. Yu , J. Xu , C. Wei , Y. Wang , H. Chen , J. Mater. Chem. A 2019, 7, 18846.

[22] B. R. Anne , S. Il Choi , Curr. Opin. Electrochem. 2023, 39, 101303.

[23] S. Huang , S. Lu , S. Gong , Q. Zhang , F. Duan , H. Zhu , H. Gu , W. Dong , M. Du , ACS Nano 2022, 16, 522. 34939416 10.1021/acsnano.1c07574

[24] M. Xie , S. Tang , B. Zhang , G. Yu , Mater. Horiz. 2023, 10, 407. 36541177 10.1039/d2mh01213h

[25] K. Chen , Z. Ma , X. Li , J. Kang , D. Ma , K. Chu , Adv. Funct. Mater. 2023, 33, 2209890.

[26] Y. Xiong , J. M. McLellan , J. Chen , Y. Yin , Z. Li , Y. Xia , J. Am. Chem. Soc. 2005, 127, 17118. 16316260 10.1021/ja056498s

[27] J. Guo , L. Gao , X. Tan , Y. Yuan , J. Kim , Y. Wang , H. Wang , Y. J. Zeng , S. Il Choi , S. C. Smith , H. Huang , Angew. Chem., Int. Ed. 2021, 60, 10942. 10.1002/anie.202100307 33751779

[28] M. Luo , Z. Zhao , Y. Zhang , Y. Sun , Y. Xing , F. Lv , Y. Yang , X. Zhang , S. Hwang , Y. Qin , J. Y. Ma , F. Lin , D. Su , G. Lu , S. Guo , NatureNature 2019, 574, 81. 10.1038/s41586-019-1603-7 31554968

[29] K. Zhang , Y. He , R. Guo , W. Wang , Q. Zhan , R. Li , T. He , C. Wu , M. Jin , ACS Energy Lett. 2022, 7, 3329.

[30] H. Yu , T. Zhou , Z. Wang , Y. Xu , X. Li , L. Wang , H. Wang , Angew. Chem., Int. Ed. 2021, 60, 12027. 10.1002/anie.202101019 33559316

[31] J. Ge , P. Wei , G. Wu , Y. Liu , T. Yuan , Z. Li , Y. Qu , Y. Wu , H. Li , Z. Zhuang , X. Hong , Y. Li , Angew. Chem., Int. Ed. 2018, 57, 3435. 10.1002/anie.201800552 29411503

[32] E. A. Nagul , I. D. McKelvie , P. Worsfold , S. D. Kolev , Anal. Chim. Acta 2015, 890, 60. 26347168 10.1016/j.aca.2015.07.030

[33] J. N. Barrows , G. B. Jameson , M. T. Pope , J. Am. Chem. Soc. 1985, 107, 1771.

[34] Y. Li , Y. Yan , Y. Li , H. Zhang , D. Li , D. Yang , CrystEngComm 2015, 17, 1833.

[35] P. Castillero , V. Rico‐Gavira , C. López‐Santos , A. Barranco , V. Pérez‐Dieste , C. Escudero , J. P. Espinós , A. R. González‐Elipe , J. Phys. Chem. C 2017, 121, 15719.

[36] H. Wang , H. Zheng , L. Ling , Q. Fang , L. Jiao , L. Zheng , Y. Qin , Z. Luo , W. Gu , W. Song , C. Zhu , ACS Nano 2022, 16, 21266. 36441949 10.1021/acsnano.2c09270

[37] K. Chen , F. Wang , X. Lu , Y. Li , K. Chu , ACS Catal 2023, 13, 9550.

[38] K. Chen , Z. Ma , X. Li , J. Kang , D. Ma , K. Chu , Adv. Funct. Mater. 2023, 33, 2209890.

[39] J. Wu , J. Fan , X. Zhao , Y. Wang , D. Wang , H. Liu , L. Gu , Q. Zhang , L. Zheng , D. J. Singh , X. Cui , W. Zheng , Angew. Chem., Int. Ed. 2022, 61, 202207512. 10.1002/anie.202207512 35762984

[40] Y. Wang , D. Sun , M. Wang , Z. Feng , A. S. Hall , J. Phys. Chem. C 2020, 124, 5220.

[41] B. Hammer , J. K. Nørskov , Surf. Sci. 1995, 343, 211.

[42] G. Kresse , J. Furthmüller , Comput. Mater. Sci. 1996, 6, 15.

[43] G. Kresse , J. Hafner , Phys. Rev. B 1994, 49, 14251. 10.1103/physrevb.49.14251 10010505

[44] G. Kresse , D. Joubert , Phys. Rev. B 1999, 59, 1758.

[45] K. Lee , É. D. Murray , L. Kong , B. I. Lundqvist , D. C. Langreth , Phys. Rev. B 2010, 82, 081101.

[46] V. Wang , N. Xu , J. C. Liu , G. Tang , W. T. Geng , Comput. Phys. Commun. 2021, 267, 108033.

