# Supplementary material for: Atomically Engineered Defect‐Rich Palladium Metallene for High‐Performance Alkaline Oxygen Reduction Electrocatalysis
Source: Adv Sci (Weinh). 2024 Aug 19;11(39):2405187. doi: 10.1002/advs.202405187 (PMC11497008; doi:10.1002/advs.202405187)
Supplement: Supplementary file 1 — Supporting Information [file ADVS-11-2405187-s001.pdf]

ADVANCED SCIENCE
Open Access

Supporting Information

for *Adv. Sci.*, DOI 10.1002/advs.202405187

Atomically Engineered Defect-Rich Palladium Metallene for High-Performance Alkaline Oxygen Reduction Electrocatalysis

Yupeng Zhao, Zhengfan Chen, Nana Ma, Weiyi Cheng, Dong Zhang, Kecheng Cao, Fan Feng, Dandan Gao, Rongji Liu, Shujun Li* and Carsten Streb**

Atomically Engineered Defect-Rich Palladium Metallene for High-Performance Alkaline Oxygen Reduction Electrocatalysis

Yupeng Zhao, Zhengfan Chen, Nana Ma, Weiyi Cheng, Dong Zhang, Kecheng Cao, Fan Feng, Dandan Gao, Rongji Liu*, Shujun Li*, Carsten Streb*

Supporting Information

Table of contents

Table of contents 1
S1 Experimental procedures 2
S2 Characterizations 3
S3 Electrochemical measurements: 4
S4 Computational details 6
S5 Zn-air battery meansurement.. 6
S6 Supplementary figures 7
S7 Reference..25
S8 Author contributions..26

S1 Experimental procedures

Chemicals and Materials: Palladium (II) acetylacetonate ($Pd(acac)_2$, 99%), 12-Molybdophosphoric acid hydrate ($H_3PMo_{12}O_{40} \cdot xH_2O$, analysis), and cyclohexane (≥ 99.9%) were purchased from Merck. Tungsten hexacarbonyl ($W(CO)_6$, 97%) and Nafion solution (D-520 dispersion 5% w/w aq. and 1-propanol) were purchased from Alfa Aesar. Potassium hydroxide (KOH, ≥85%), ethanol (CH_3CH_2OH, absolute), commercial Pt/C (20 wt%), and commercial Pd/C (10 wt%), and N, N-Dimethylformamide (DMF, 99.5%) were purchased from Fisher Scientific. Acetic acid (CH_3COOH, 99.5%) was purchased from VWR. Cetyltrimethylammonium bromide (CTAB, $C_{19}H_{42}BrN$), citric acid (CA, $C_6H_8O_7$, ≥ 99.5 %), polyvinyl pyrrolidone (PVP, Mw = 55 000) were purchased from Sigma-Aldrich. Vulcan XC-72 carbon was purchased from BC Berlin Catalysts GmbH. All chemicals were used in this experiment without further purification.

Synthesis of D-Pd M and Pd M:

The defect-rich Pd metallene (**D-Pd M**) was synthesized by a simple wet-chemical method. Typically, 42 mg of $H_3PMo_{12}O_{40} \cdot xH_2O$ was dissolved in 10 mL of DMF solution followed by deaeration with Ar for 15 min. Then the solution was irradiated under UV light (Wavelength = 254 nm) for 30 min to get solution A. In addition, 10.0 mg of $Pd(acac)_2$ and 20 mg of $W(CO)_6$ were added into a 25 mL bottle containing 8 mL of DMF and then sonicated for half an hour to yield a homogeneous solution (B). Afterward, solution A was added to solution B and was placed in an oil bath at 50 ℃ for 6 h. After cooling to room temperature, the black colloidal product was collected by centrifugation and washed six times with a mixture of ethanol and cyclohexane ($v_{ethanol}$ /$v_{cyclohexane}$ = 1:1). The final product was dispersed in cyclohexane for further use.

The hexagonal Pd metallene (Pd M) for comparison were prepared according to a previous approach with slight modifications.[1,2] 16 mg of $Pd(acac)_2$, 90 mg of CA, 60 mg of CTAB, and 30 mg of PVP were added into 10 mL of DMF and stirred for 1 h to get a homogeneous orange-red solution. The obtained solution was then transferred into a 25 mL vial and 100 mg of $W(CO)_6$ was added to the vial under N_2 atmosphere. The vial was capped and heated at 80 °C for 1 h. After the reaction, Pd nanosheets were collected by centrifugation using enough acetone and then redispersed in acetone for further use.

S2 Characterizations

Powder X-ray diffraction (XRD): Bruker D2 Phaser equipment, with Cu Kα radiation (λ = 1.5406 Å).

Transmission electron microscopy (TEM): Tecnai G2 Spirit operated at 120 kV (Imaging).

High-resolution transmission electron microscopy (HRTEM) and energy-dispersive X-ray spectroscopy (EDX): Jeol F200 equipped with a SuperX EDX detector, operated at 200 kV (Imaging and diffraction pattern).

Inductively Coupled Plasma Optical Emission Spectroscopy (ICP-OES): Agilent 5800 VDV ICP-OES (optical emission spectrometer) with the automatic sampler SPS 4.

X-ray photoelectron spectroscopy (XPS): Monochromatized Al Kα exciting X-radiation using a PHI Quantera SXM system. The binding energies were calibrated based on C1s (284.8 eV).

Ultraviolet - visible spectroscopy (UV-vis): Cary 3500 UV-Vis Spectrophotometer equipped with a Xenon flash lamp (250 Hz). Measurements were performed in standard 1 cm cuvettes.

Atomic force microscopy (AFM): Bruker Dimension Icon.

X-ray absorption spectroscopy (XAS) experiment and data processing: Pd K-edge, W L_3-edge, and Mo L_3-edge X-ray absorption fine structure (XAFS) analyses were performed with Si (111) crystal monochromators at the BL14W Beam line at the Shanghai Synchrotron Radiation Facility (SSRF) (Shanghai, China). Before the analysis at the beamline, samples were placed into aluminum sample holders and sealed using Kapton tape film. The XAFS spectra were recorded at room temperature using a 4-channel Silicon Drift Detector (SDD) Bruker 5040. Pd K-edge and W L_3-edge extended X-ray absorption fine structure (EXAFS) spectra were recorded in transmission mode. Mo L_3-edge extended X-ray absorption fine structure (EXAFS) spectra were recorded in fluorescence mode. Negligible changes in the line shape and peak position of Pd K-edge, W L_3-edge, and Mo L_3-edge XANES spectra were observed between two scans taken for a specific sample. The spectra were processed and analyzed by the software code Athena.

S3 Electrochemical measurements:

The as-prepared **D-Pd M** and Pd M catalysts were loaded on carbon black (Vulcan XC-72) before electrochemical tests. Typically, the **D-Pd M** (2 mg) was mixed with carbon black (8 mg) in cyclohexane (10 mL) under ambient sonication for 1 h. After stirring for another 12 h, the product was collected by centrifugation and washed three times with ethanol. Afterward, the product was treated in pure acetic acid (10 mL) at 70 °C for 2 h to remove excess impurities and excess surfactants for further use. At the end, **D-Pd M**/C was collected by centrifugation, washed three times with ethanol, and dried at 60°C for 3 hours. Pd M/C was prepared by the same method.

To prepare the catalyst inks, the as-prepared **D-Pd M**/C (1 mg) was re-dispersed in a solution containing 15 μL DI water, 980 μL ethanol, and 5 μL Nafion solution by at least 30 min sonication in an ice-water bath to form a catalyst ink. Then the **D-Pd M**/C catalyst ink was dipped on the electrode with a controllable amount of **D-Pd M** catalyst being 10 $\mu g \cdot cm^{-2}$. Likewise, the Pd M/C was processed in the same way. The controllable amount of commercial Pd/C, and commercial Pt/C were 15 $\mu g \cdot cm^{-2}$ to reach the well-defined limited current density. The loading amount on the electrode of prepared catalysts was determined by ICP-OES.

Electrochemical tests were conducted using a three-electrode system on an Ametek electrochemical workstation (channel PMC 1000) equipped with an RRDE setup (Pine, USA). A glassy carbon rotating disk electrode (GC RDE, diameter: 5 mm, area: 0.196 cm^2), a saturated calomel electrode (SCE), and a graphite rod were used as the working electrode, reference electrode, and counter electrode, respectively. All potentials in this study were reported on a reversible hydrogen electrode (RHE) scale according to the Nernst equation ($E_{RHE} = E_{SCE} + E^0_{SCE} + 0.059\ V \times pH$). The cyclic voltammetry (CV) curves were recorded in N_2-saturated 0.1 M KOH at a scan rate of 50 $mV \cdot s^{-1}$. ORR linear sweep voltammetry (LSV) was recorded in O_2-saturated 0.1 M KOH at 20 $mV \cdot s^{-1}$ and a rotating rate of 1600 rpm. It is worth noting that the ORR LSV curves have been corrected in the N_2-saturated 0.1 M KOH to eliminate the interference of double-layer capacity. The current densities ($\boldsymbol{j}$) were normalized regarding the geometric area of the GC RDE. Notably, all ORR LSV curves were iR-corrected (95%) before applying the K-L equation: $\boldsymbol{I}_k = (\boldsymbol{I}_d \times \boldsymbol{I})/(\boldsymbol{I}_d - \boldsymbol{I})$, where $\boldsymbol{I}_k$, $\boldsymbol{I}_d$, and $\boldsymbol{I}$ represent the kinetic, diffusion limiting, and measured current, respectively.

The electrochemical active surface areas (ECSAs) of all the catalysts were calculated using the underpotentially deposited H (H_{upd}) and Cu stripping. The underpotentially deposited H (H_{upd})

data was obtained in the N_2-saturated 0.1 M $HClO_4$. The peaks of H_{upd} were recorded by cyclic voltammetry (CV) with a scan rate of 50 $mV \cdot s^{-1}$ to calculate the ECSAs. In the calculation, we used a charge density of 210 $\mu C \cdot cm^{-2}$ for one monolayer of hydrogen coverage on commercial Pt/C and Pd/C, and a charge density of 240 $\mu C \cdot cm^{-2}$ (a value for the (111) surface) for one monolayer of hydrogen coverage on D-Pd M/C and Pd M/C. For the Cu stripping experiments, an Ar-saturated solution containing 2 mM $CuSO_4$ and 0.05 M H_2SO_4 was used as the electrolyte. The potential was first held at 0.3 V versus RHE for 100 s to form a Cu_{upd} monolayer, and then CVs were obtained from 0.3 V to 1.0 V at 20 $mV \cdot s^{-1}$. The ECSAs were calculated by subtracting the background CVs that were collected in Ar-saturated 0.05 M H_2SO_4 at 20 $mV \cdot s^{-1}$, assuming a charge density of 470 $\mu C \cdot cm^{-2}$.

The electron transfer number (n) and the hydrogen peroxide yield (***H₂O₂***%) were tested by a rotating Pt ring - GC disk electrode (RRDE) device (Pine, USA), where the surface area of the GC disk and Pt ring are 0.2475 cm^{-2} and 0.1866 cm^{-2}, respectively. The disk electrode was conducted LSV scan, and the ring electrode potential was set to 1.4 V vs. RHE. The $\boldsymbol{H_2O_2}$% and n were calculated by the following equations:

$$\boldsymbol{H_2O_2}\% = 200 \times \frac{\frac{I_r}{N}}{\frac{I_r}{N}+I_d}$$

$$n = 4 \times \frac{I_d}{\frac{I_r}{N}+I_d}$$

where $\boldsymbol{I}_d$ is the disk current, $\boldsymbol{I}_r$ is the ring current, and N is the ring collection efficiency (N=0.37). In addition, the electron transfer number during the ORR process of **D-Pd M** was also determined by applying different rotating rates to the Koutecky-Levich equation:

$$\frac{1}{j} = \frac{1}{j_K} + \frac{1}{j_L} = \frac{1}{B\omega^{1/2}} + \frac{1}{j_L}$$

$$B = 0.2nFC_0D_0^{2/3}v^{-1/6}$$

where j, j_k, and j_L represent measured current density, the kinetic and limiting current densities the kinetic, respectively. F is the Faraday constant (96485 C mol^{-1}), C_0 is the bulk concentration of O_2 (1.26×10^{-6} $mol \cdot cm^{-3}$), D_0 is the diffusion coefficient of O_2 in 0.1 M KOH (1.93×10^{-5} $cm^2 \cdot s^{-1}$), and v is the kinetic viscosity (0.01 $cm^2 \cdot s^{-1}$). The rotation speed is expressed in rpm.

Accelerated durability tests (ADTs) were conducted by cycling between 0.6 V and 1.0 V vs. RHE at 200 mV/s for 5,000 cycles and 10,000 cycles in 0.1 M O_2-saturated KOH solution at 1600 rpm.

S4 Computational details

All DFT calculations in the present work were performed using the Vienna Ab initio Simulation Package (VASP)[3,4] with the Perdew-Burke-Ernzerhof (PBE)[3] functional. The ion-electron interactions were modeled using the projector-augmented wave (PAW) method[5]. The plane wave cutoff energy was set to 400 eV in all calculations. The convergence criteria were set to be 10-5 eV and 0.01 eV/Å for wavefunction and geometry optimization, respectively. A Γ-centred Monkhorst–Pack k-point mesh with a size of 3×3×1was applied for all supercells. The vacuum region was set to 15 Å. The DFT-D3 scheme of dispersion correction was employed to describe van der Waals interactions[6]. The electronic density of the structure was processed using VASPKIT[7].

S5 Zn-air battery meansurement

The zinc-air battery was measured in a labmade battery mode, consisting of an anode and a cathode. The anode is a polished Zn foil (with a thickness of 0.5 mm), the air cathode is the catalyst-loaded carbon paper (ThermoFisher Scientific), and the electrolyte is a 6 M KOH solution containing 0.2 M $Zn(CH_3COO)_2$. The catalyst inks were prepared with the same method we mentioned above but at a concentration of 10 mg·mL^{-1}. The catalyst-loaded carbon paper was prepared by spreading as-prepared catalyst ink onto carbon paper and drying at room temperature. The mass loading of 20% **D-Pd M**/C and 20% Pt/C catalysts is 130 μg·cm^{-2}, and the mass loading of Pd/Pt is 26 μg·cm^{-2}. The polarization curves were performed at the CHI 760E electrochemistry station. Galvanostatic cycling tests were collected at the **LAND CT2003A** multi-channel battery testing system. When testing the specific capacity of zinc-air potential, the discharge current was set at 15 mA and the cut-off voltage was 0.5 V. The charge and discharge current density was set at 15 mA, the discharge cut-off voltage was set to 0.2 V, and the charge cut-off voltage was set to 1. 85 V.

S6 Supplementary figures

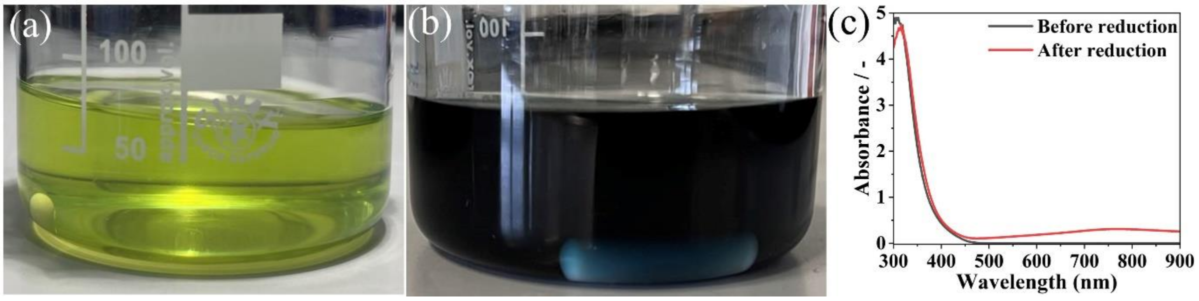

Figure S1. The optical photographs of PMo_{12} in DMF solution before (a) and after (b) reduction, and corresponding UV-visible spectra after being diluted to 1/10 of original concentration.

The color changes from bright yellow to dark blue and newly appeared broad in reduced solution indicate the reduction of PMo_{12} clusters to heteropoly blue. Approximately 5.2% of PMo_{12} underwent reduction to form heteropoly blue,[8,9] according to the Beer-Lambert law: $A = \varepsilon bc$; A is absorbance, ε is molar absorptivity (here: 2600 $M^{-1}\cdot cm^{-1}$), b is length of light path (here: 1 cm), and c is concentration, respectively.

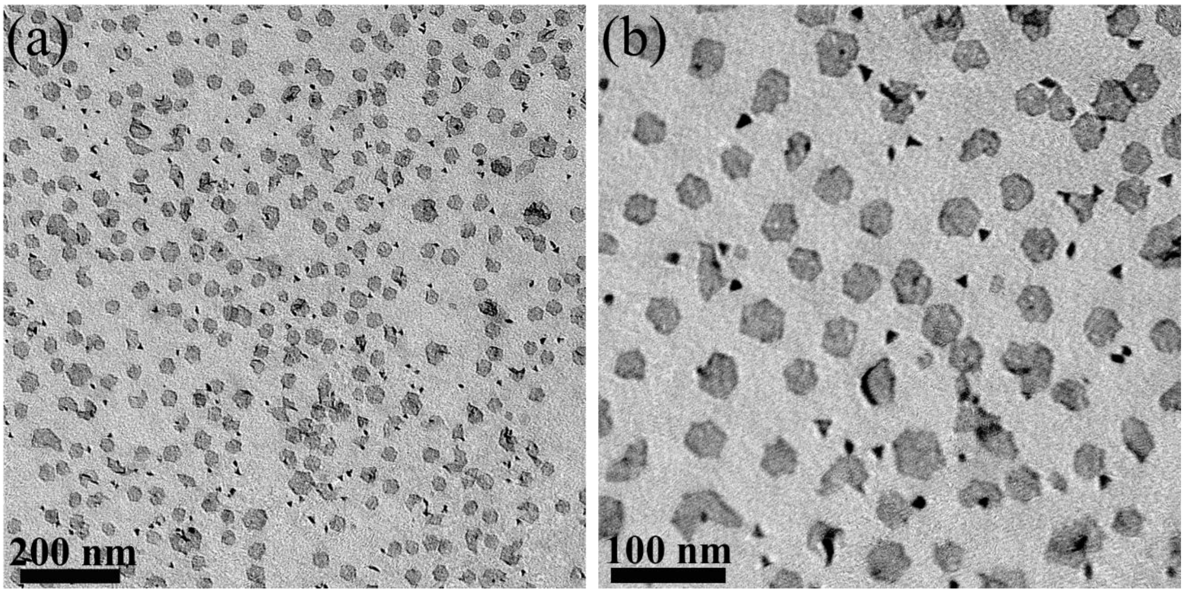

Figure S2. TEM images of Pd M at low (a) and (high) magnification.

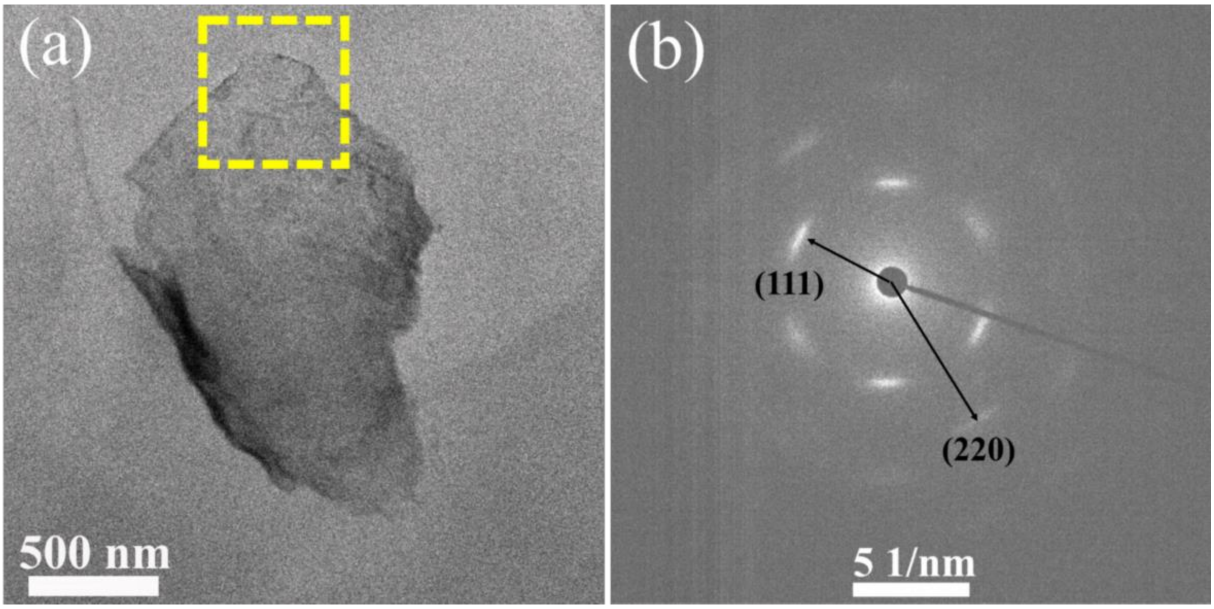

Figure S3. (a) TEM image and (b) the corresponding selected area electron diffraction (SAED) pattern in selected area in (a) of **D-Pd M**.

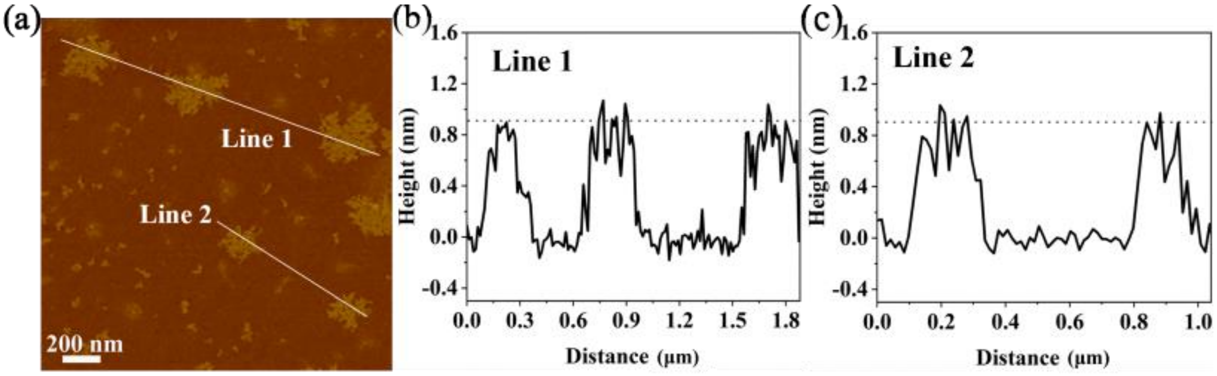

Figure S4. Atomic force microscopy (AFM) image (a) and corresponding height profiles (b and c) of **D-Pd M**.

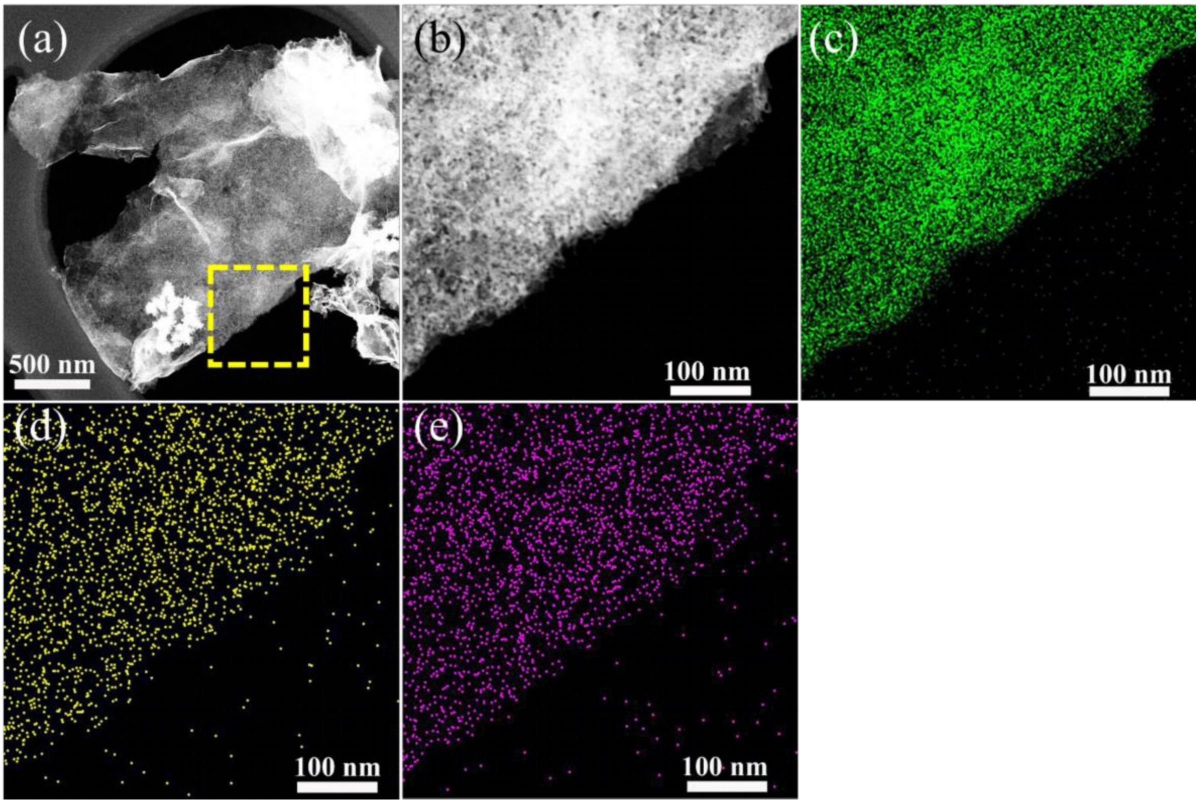

Figure S5. (a) HAADF-STEM image, (b) the magnification of selected area (in a, yellow square), (c-d) and the corresponding elemental mapping of Pd, Mo, and W elements of the **D-Pd M**.

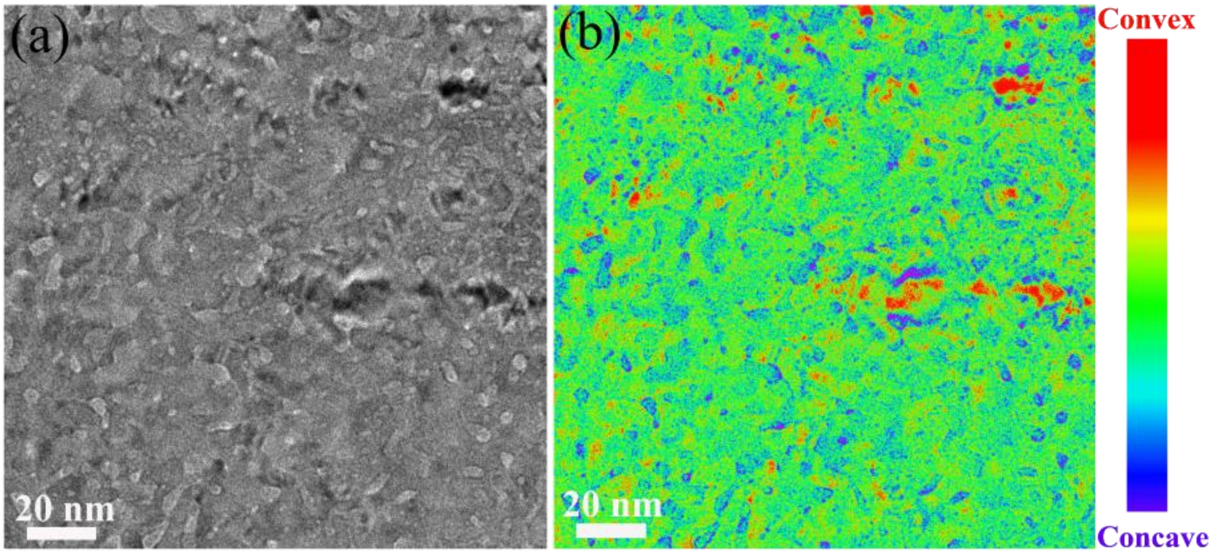

Figure S6. (a) TEM images and (b) corresponding false color mode of **D-Pd M**.

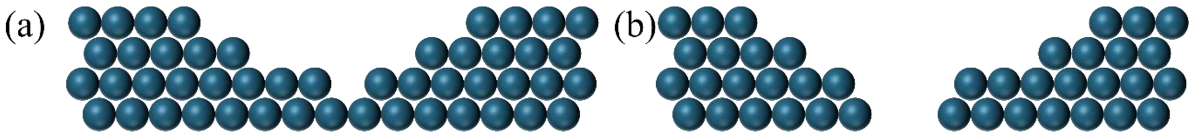

Figure S7. Schematic of pores (a) and concave (b) defects from side view.

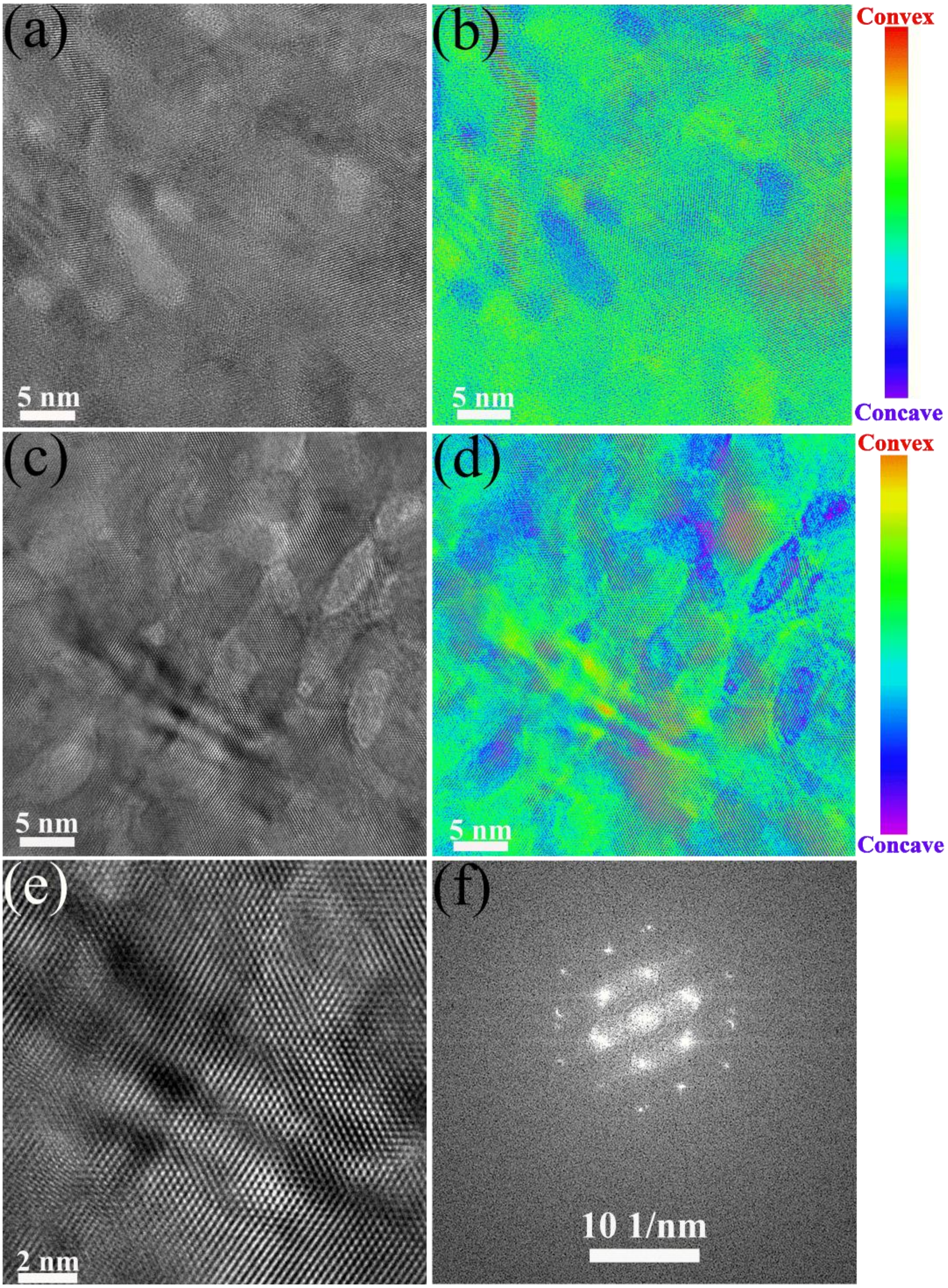

Figure S8. HRTEM images of **D-Pd M**. (a, c, and e) crude images, (b and d) temperature mode of corresponding images, in which dark blue represents concave sites and bright orange represents convex sites. (f) fast Fourier transform pattern of (e).

Table S1. EXAFS fitting parameters at the Mo&Pd *K*-edge and W L_3-edge for **D-Pd M**.

Sample	Shell	CN^a	R(Å)^b	σ^2(Å^2)^c	ΔE_0(eV)^d	R factor
Pd						
Pd foil	Pd-Pd	12*	2.737±0.002	0.0055±0.0002	3.4±0.2	0.0020
PdO	Pd-O	4.0±0.2	2.017±0.001	0.0023±0.0006	7.9±0.3	0.0042
	Pd-Pd	3.7±0.8	3.018±0.001	0.0089±0.0017	1.5±0.9	
	Pd-Pd	7.7±0.8	3.388±0.001	0.0124±0.0001	2.3±0.7	
**D-Pd M**	Pd-Pd/Mo/W	10.7±1.0	2.738±0.005	0.0060±0.0006	6.0±0.7	0.0062
W						
W-foil	W-W	8*	2.734±0.006	0.0037±0.0007	5.3±1.0	0.0049
	W-W	6*	3.160±0.012	0.0043±0.0010	8.4±1.7	
WO_2	W-O	4.0±0.7	2.013±0.001	0.0015±0.0021	11.4±1.0	0.0081
**D-Pd M**	W-O	1.4±0.3	1.779±0.001	0.0070±0.0014	-7.9±1.0	0.0062
	W-O	2.8±0.4	2.111±0.001			
	W-Pd	6.3±0.8	2.605±0.001	0.0189±0.0011	2.0±0.6	
	W-O	15.9±1.8	3.499±0.001			
Mo						
Mo foil	Mo-Mo	8*	2.724±0.003	0.0041±0.0003	4.9±0.5	0.0040
	Mo-Mo	6*	3.150±0.005	0.0040±0.0004	7.5±1.0	
MoO_2	Mo-O	6.0±0.3	1.999±0.001	0.0033±0.0006	2.8±0.3	0.0035
	Mo-Mo	3.1±0.6	2.569±0.001	0.0105±0.0017	8.8±0.8	
	Mo-Mo	1.9±0.2	3.087±0.001	0.0040±0.0001	-11.1±0.5	
	Mo-Mo	6.2±0.3	3.642±0.001			
	Mo-Mo	7.4±1.6	4.058±0.001	0.0130±0.0001	0.8±0.1	
MoO_3	Mo-O	2.0±0.2	1.685±0.001	0.0037±0.0006	4.5±0.9	0.0080
	Mo-O	2.7±0.3	1.975±0.001		14.0±0.1	
	Mo-O	3.1±0.4	2.286±0.001	0.0095±0.0001		
	Mo-Mo	6.3±1.1	3.430±0.001	0.0086±0.0006	-	
	Mo-Mo	15.4±1.9	3.625±0.001		12.0±0.4	
	Mo-Mo	2.5±0.4	4.062±0.001	0.0051±0.0001	13.3±1.3	
**D-Pd M**	Mo-O	1.4±0.2	1.745±0.001	0.0023±0.0009	8.5±0.8	0.0070
	Mo-O	0.4±0.2	2.044±0.001			
	Mo-O	0.5±0.2	2.429±0.001	0.0046±0.0014	-3.0±1.2	
	Mo-Pd	0.9±0.2	2.681±0.001			

$^a CN$, coordination number; $^b R$, the distance to the neighboring atom; $^c \sigma^2$, Debye-Waller factor, the Mean Square Relative Displacement (MSRD); $^d \Delta E_0$, inner potential correction; R factor indicates the goodness of the fit. S_0^2 was fixed to 0.992, 0.812, and 0.958, according to the experimental EXAFS fit of Mo foil, Pd foil, and W foil by fixing CN as the known crystallographic value. * This value was fixed during EXAFS fitting, based on the known structure of Mo, Pd, and W. Fitting range: $3.0 \leq k$ (/Å) ≤ 14.0 and $1.0 \leq R$ (Å) ≤ 3.3 (Mo foil); $3.0 \leq k$ (/Å) ≤ 12.0 and $1.1 \leq R$ (Å) ≤ 3.9 (MoO2); $3.0 \leq k$ (/Å) ≤ 13.0 and $1.0 \leq R$ (Å) ≤ 4.1 (MoO_3); $3.0 \leq k$ (/Å) ≤ 13.0 and $1.0 \leq R$ (Å) ≤ 3.0 (D-Pd M_Mo); $3.0 \leq k$ (/Å) ≤ 13.0 and $1.0 \leq R$ (Å) ≤ 2.9 (Pd foil); $3.0 \leq k$ (/Å) ≤ 12.0 and $1.0 \leq R$ (Å) ≤ 3.6 (PdO); $3.0 \leq k$ (/Å) ≤ 13.0 and $1.9 \leq R$ (Å) ≤ 3.0 (D-Pd M_Pd); $3.0 \leq k$ (/Å) ≤ 12.0 and $1.0 \leq R$ (Å) ≤ 3.5 (W foil); $3.0 \leq k$ (/Å) ≤ 11.0 and $1.2 \leq R$ (Å) ≤ 2.0 (WO_2); $3.0 \leq k$ (/Å) ≤ 10.0 and $1.0 \leq R$ (Å) ≤ 3.8 (D-Pd M_W). A reasonable range of EXAFS fitting parameters: $0.700 < S_0^2 < 1.000$; $CN > 0$; $\sigma^2 > 0$ Å^2; $|\Delta E_0| < 15$ eV; R factor < 0.02.

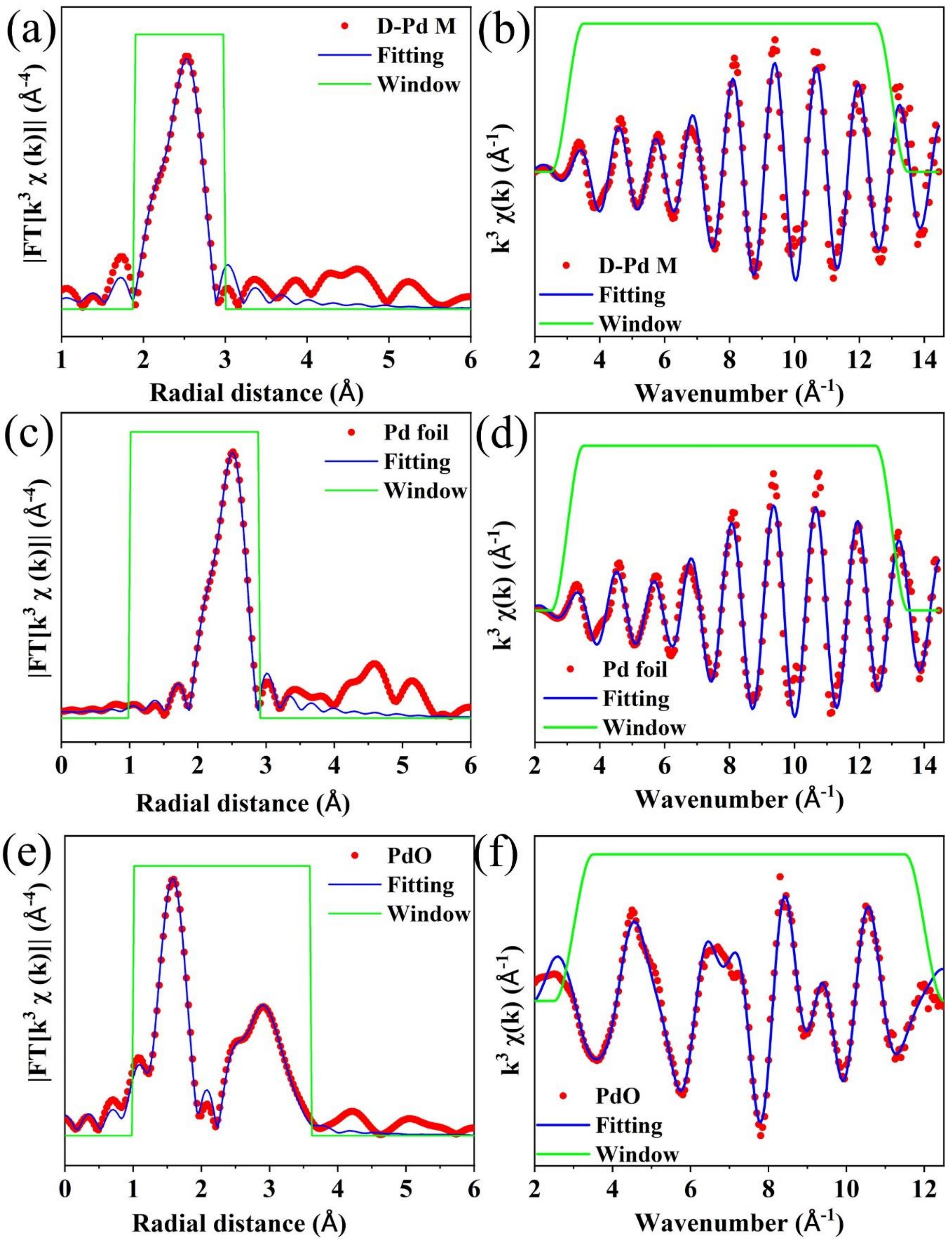

Fig. S9. R space and k space fitting results of Pd K-edge. (a and b) **D-Pd M**, (c and d) Pd foil, and (e and f) PdO. The dots or lines in red, blue, and green represent raw data, fitting results and FT fitting range windows.

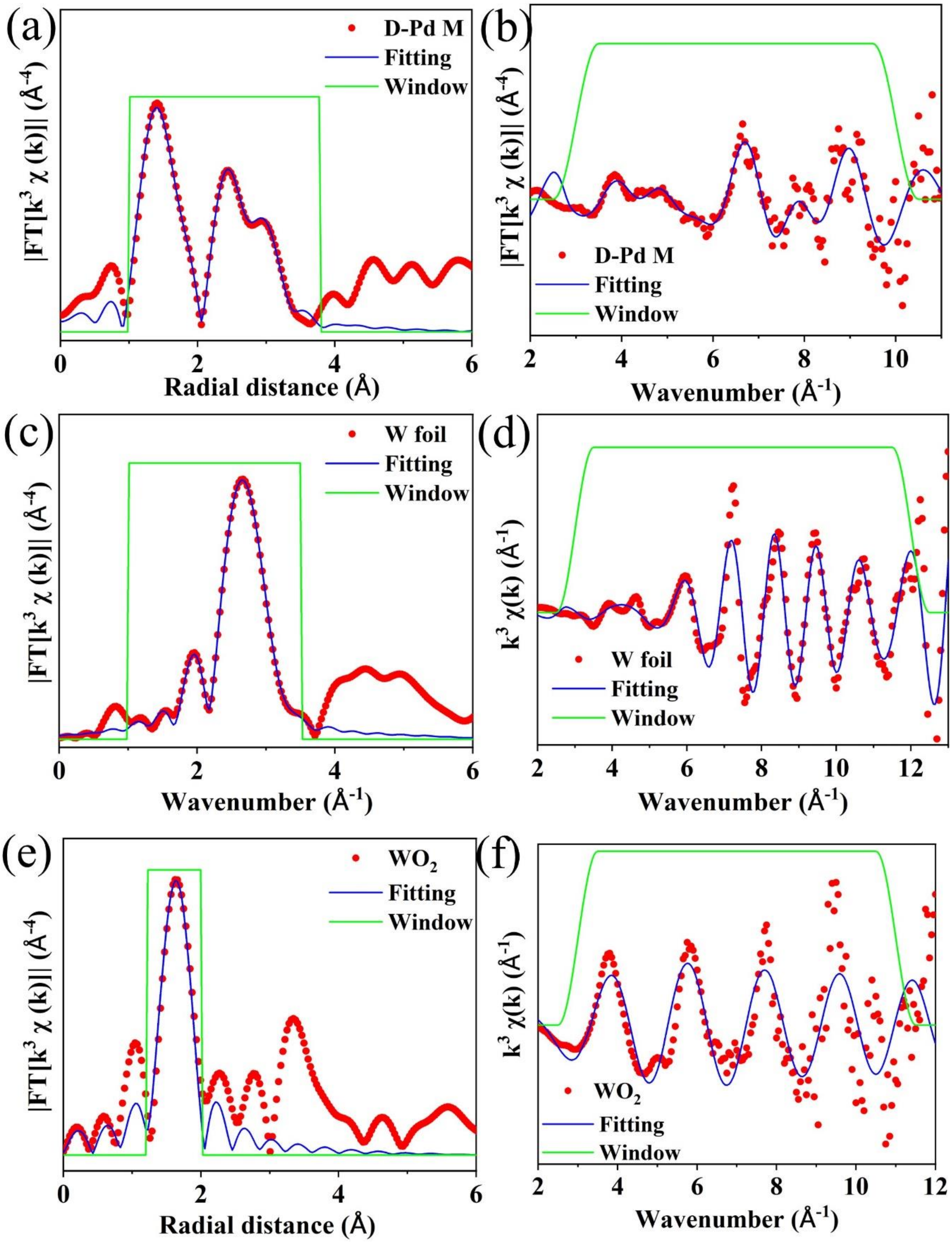

Fig. S10. R space and k space fitting results of W L-edge. (a and b) **D-Pd M**, (c and d) W foil, and (e and f) WO_2. The dots or lines in red, blue, and green represent raw data, fitting results and FT fitting range windows.

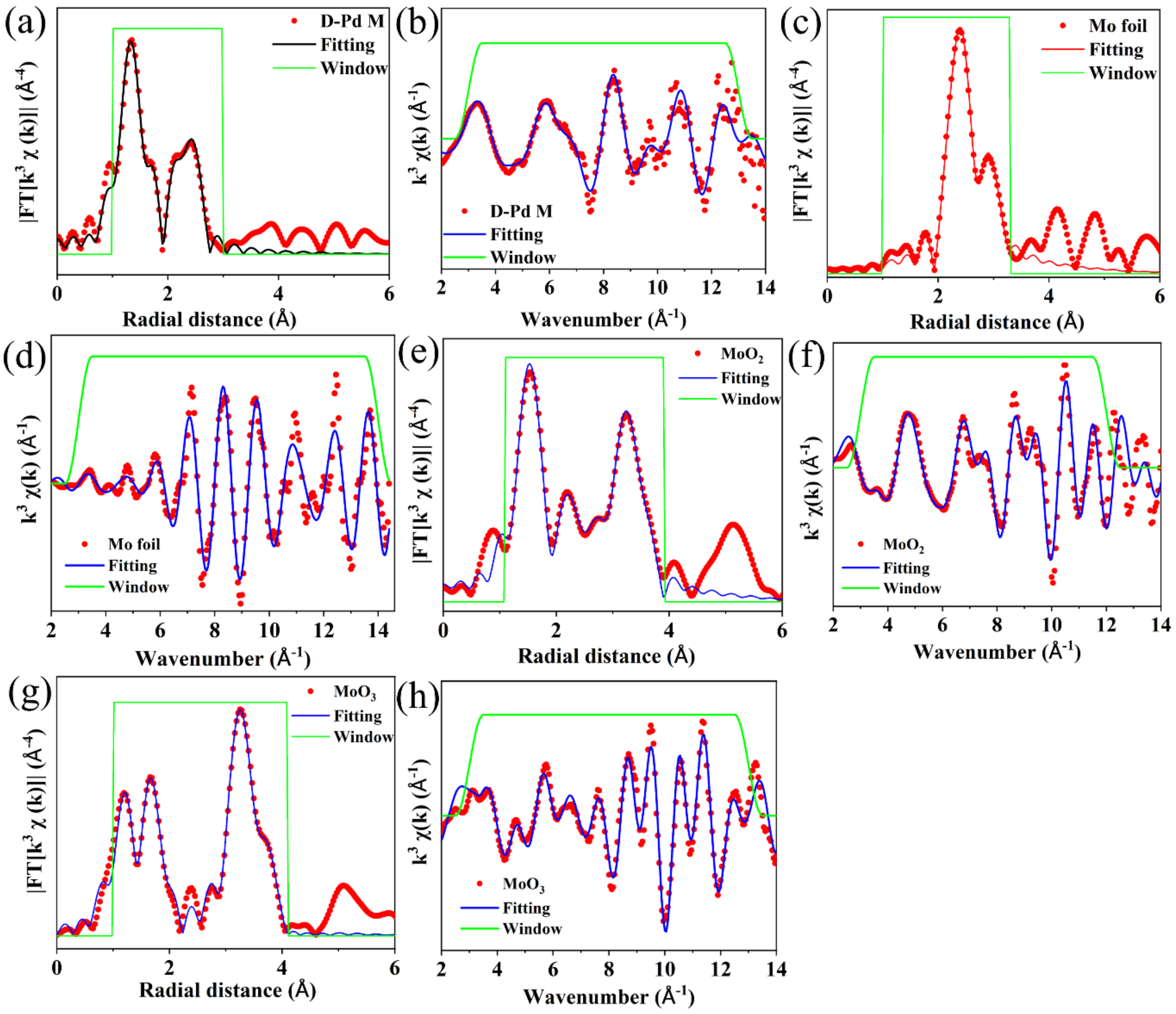

Fig. S11. R space and k space fitting results of Mo K-edge. (a and b) **D-Pd M**, (c and d) Mo foil, (e and f) MoO_2, and (g and h) MoO_3. The dots or lines in red, blue, and green represent raw data, fitting results and FT fitting range windows.

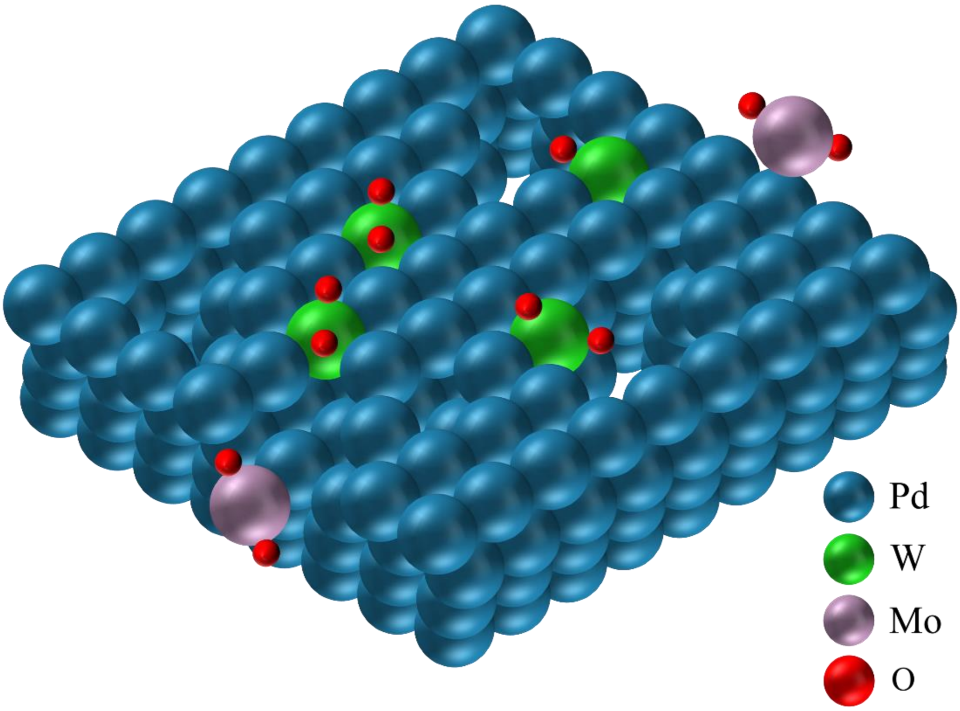

Figure S12. Schematic of MoO_x and WO_x doping. W atoms substitute the position of Pd, but Mo just dispersed on the edge of Pd.

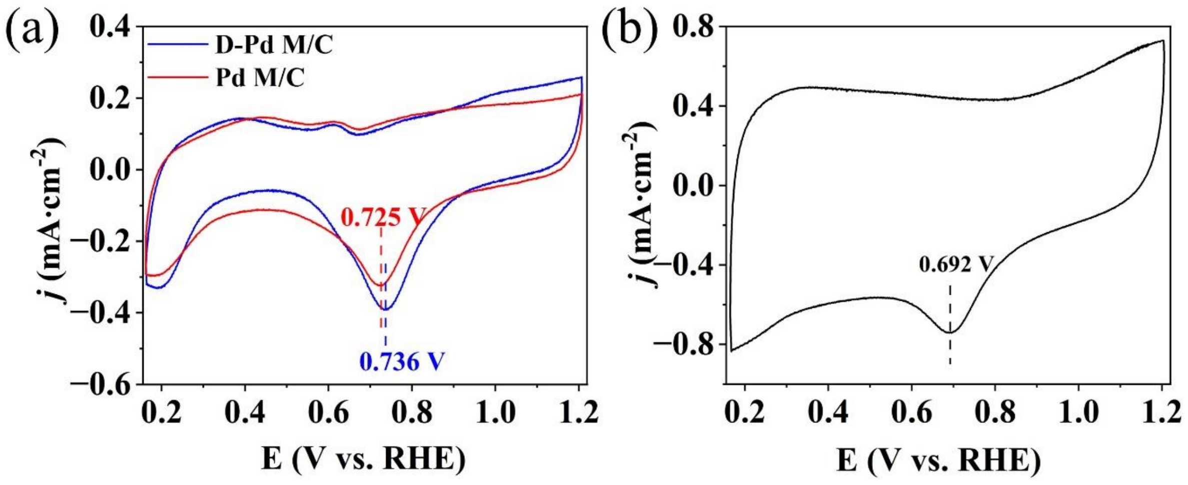

Figure S13. CV curves of (a) **D-Pd M**/C, Pd M/C, and (b) Pd/C in N_2-saturated 0.1 M KOH solution at a scan rate of 50 mV·s^{-1}.

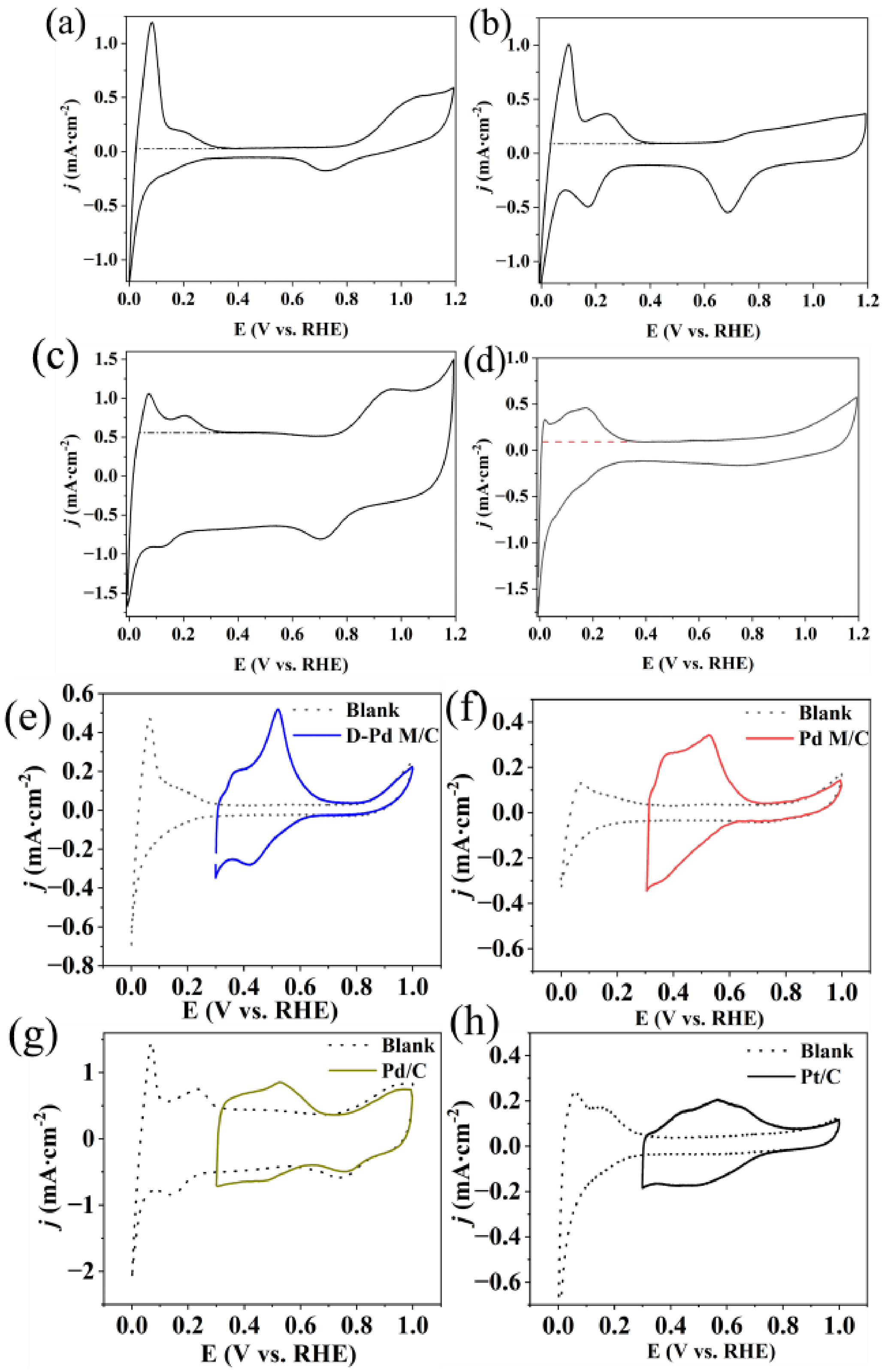

Figure S14. CVs of (a) **D-Pd M**/C, (b) Pd M/C, (c) Pd/C, and (d) Pt/C in N_2-saturated 0.1 M $HClO_4$ at a scan rate of 50 mV·s^{-1} (e-h) CVs and Cu stripping voltammograms of **D-Pd M**/C, Pd M/C, Pd/C, and Pt/C.

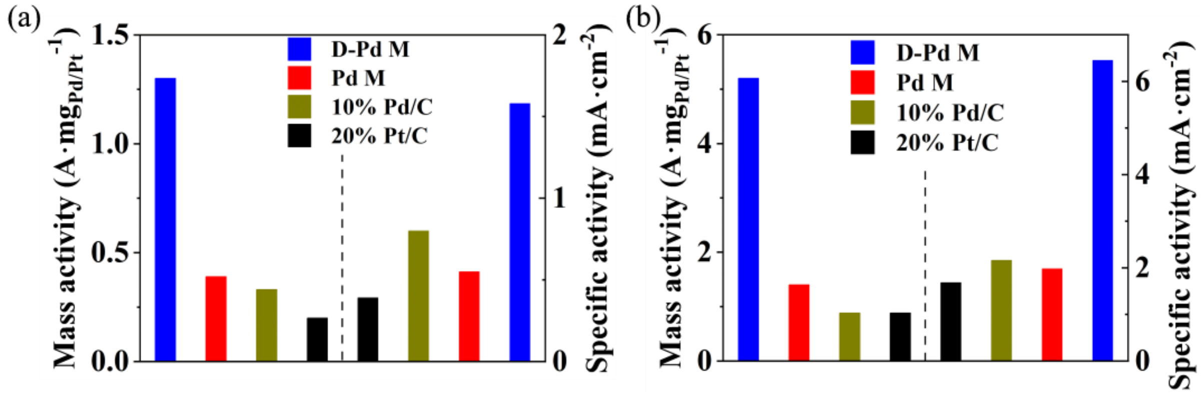

Figure S15. MAs and SAs of **D-Pd M**/C, Pd M/C, Pd/C, and Pt/C at (a) 0.9 V vs. RHE and (b) 0.85 V vs. RHE.

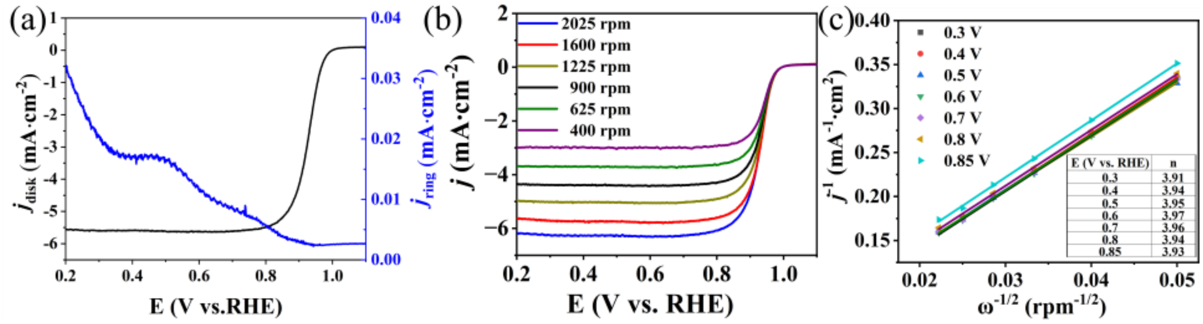

Figure S16. (a) Ring and disk current density of **D-Pd M** in O_2-saturated 0.1 M KOH solution, potential applied on ring was set 1.4 V vs. RHE, (b)LSV curves of **D-Pd M**/C at different scan rates and (c) corresponding K–L plot and electron transfer numbers (insert).

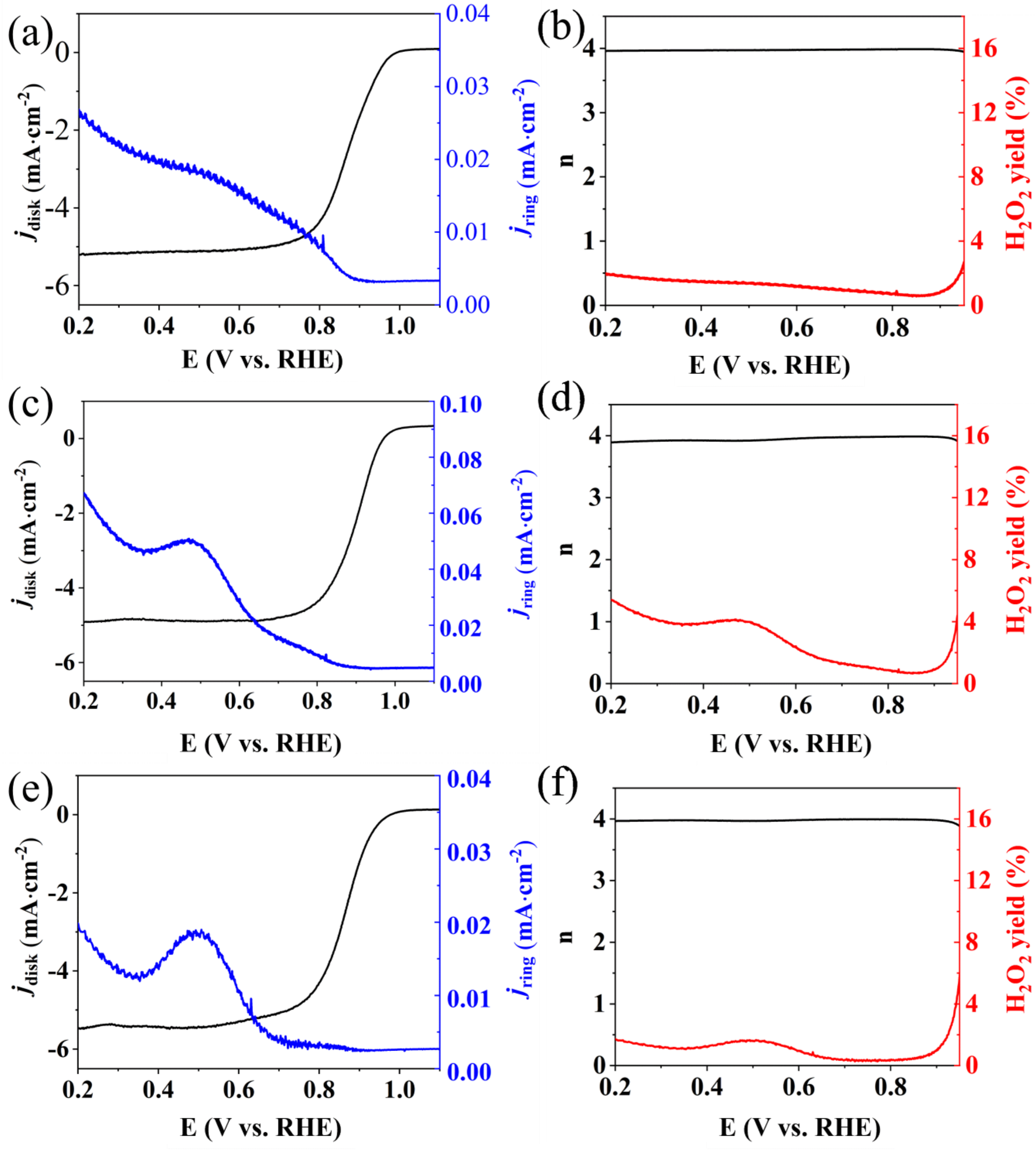

Figure S17. Ring and disk current density of (a) Pd M/C, (c) Pd/C, and (e) Pt/C in O_2-saturated 0.1 M KOH solution, potential applied on ring was set 1.4 V vs. RHE. The electron transfer number and H_2O_2 yield of (b) Pd M/C, (d) Pd/C, and (f) Pt/C.

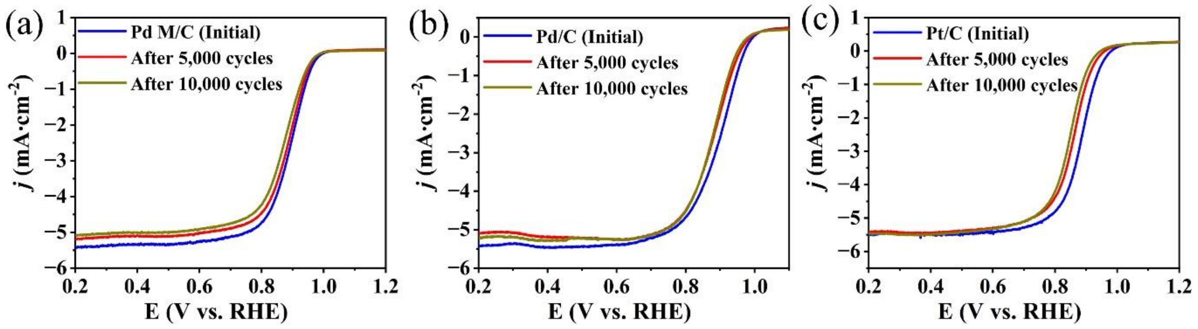

Figure S18. LSVs of (a) Pd M/C, (c) Pd/C, and (e) Pt/C in O_2-saturated 0.1 M KOH solution before and after 5,000 and 10,000 CV scans.

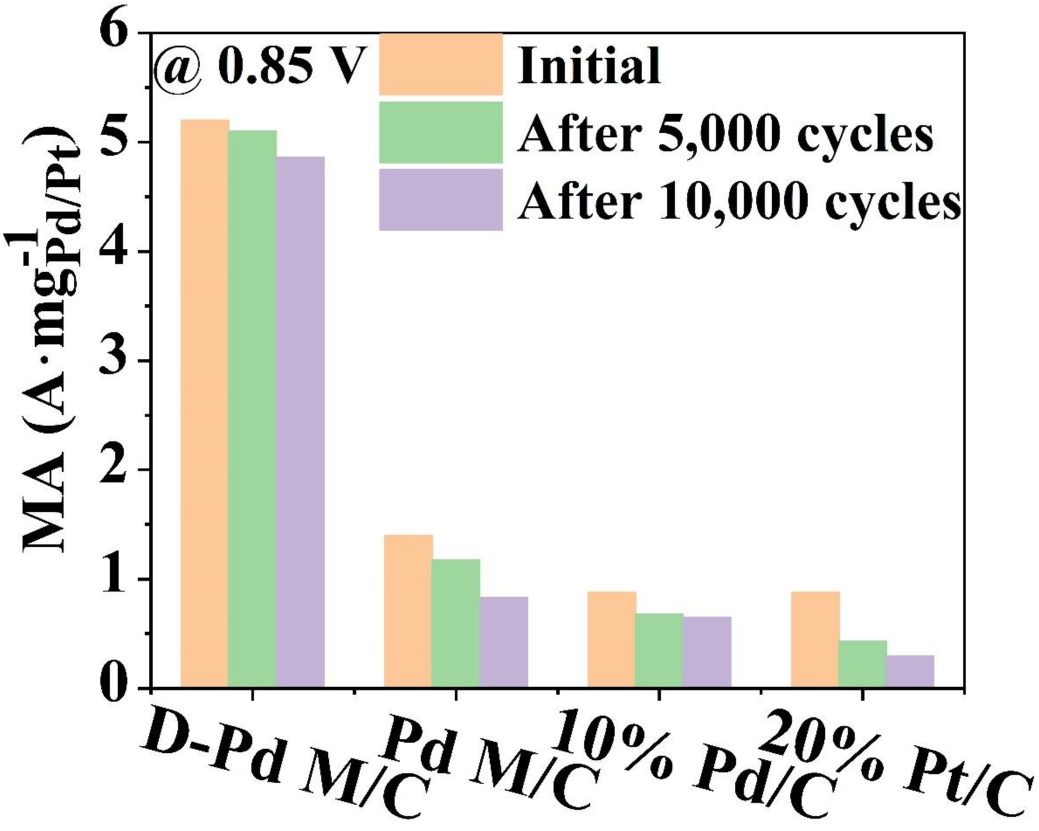

Figure S19. MAs of **D-Pd M**/C, Pd M/C, Pd/C, and Pt/C at 0.85 V vs. RHE before and after 5,000 and 10,000 cycles

Table S2. Comparison of electroactivity of recent reported representative Pd-based catalysts for alkaline ORR

Samples	Half-wave potential (V vs. RHE)	Specific activitiy (mA·cm^{-2} at 0.9 V)	Mass activitiy (A·mg_{Pd}^{-1} at 0.9 V)	Reference
**D-Pd M**	**0.93**	**6.13**	**1.3**	This work
Pd M	0.87	1.86	0.39	This work
PdCu Metallene	0.943	1.67	0.905	[10]
Defect-Rich Pd Metallene	0.90	1.336	0.892	[11]
$Pd_9Pt_1Ni_1$	0.928	1.22	0.29	[12]
Fe−Pd UPM	0.914	0.89	0.736	[13]
PdMo bimetallene	0.95	11.64	16.37	[14]
Pd_3Pb/PdTetragonal Nanosheets	0.9	1.31	0.57	[15]
Pd3Pb Nanosheet	0.91	0.989	0.697	[16]
Pd@PEI-EDA metallene	0.968	1.3	0.93	[17]
N–Pd MNRs	0.95	1.29	0.68	[18]

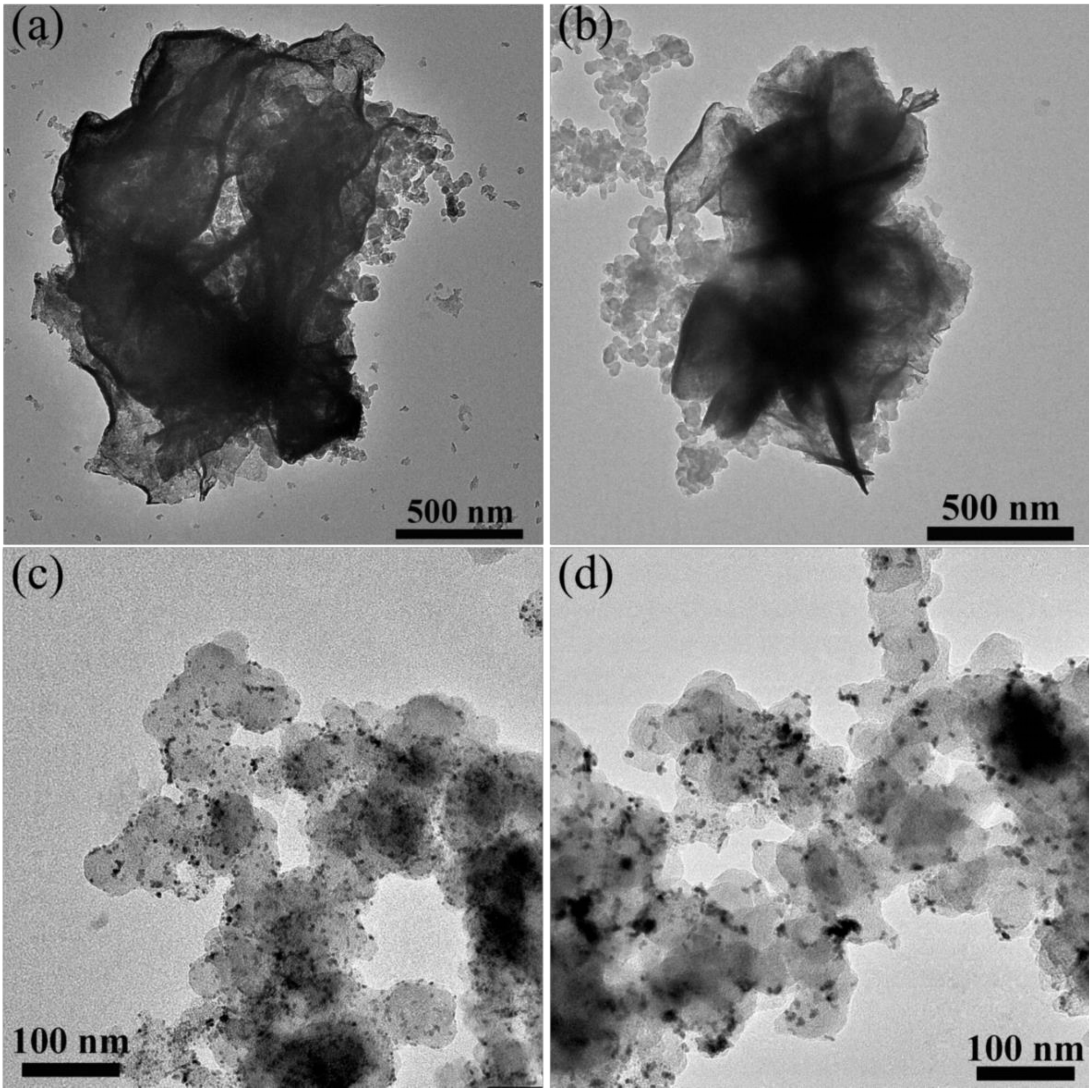

Figure S20. The morphologies of **D-Pd M** (a, b) and Pt/C (c, d) before and after ADT test.

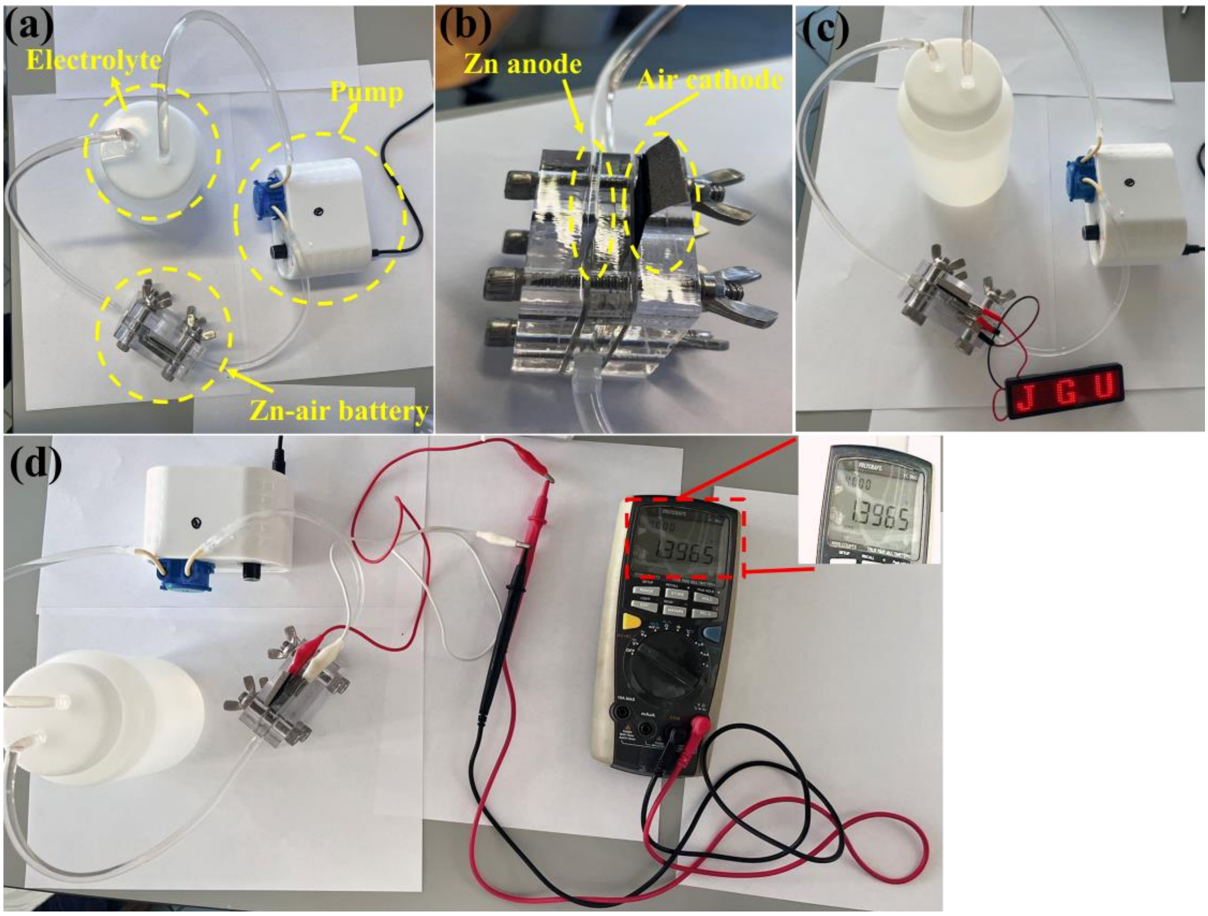

Figure S21. Photographs of the whole Zn-air battery system (a) and Zn-air battery (b), (c) red LED (the operating voltage only > **1** V) powered by **D-Pd M** based Zn-air battery, and (c) an open-circuit voltage of 1.397 V obtained with **D-Pd M** based Zn-air battery.

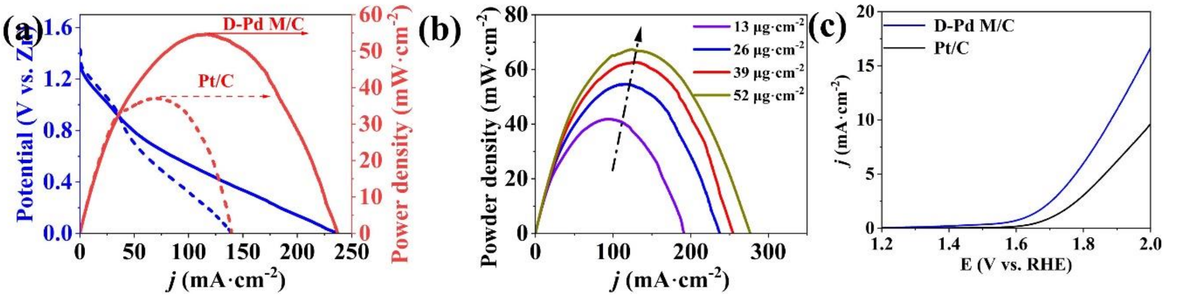

Figure S22. (a)Polarization and corresponding power density curves of **D-Pd M**/C (line) and Pt/C (dashes), (b) power density curves of **D-Pd M**/C at different loading, (c) OER polarization curves of **D-Pd M**/C and Pt/C in O_2-saturated 0.1 M KOH at a scan rate of 5 $mV{\cdot}s^{-1}$.

Table S3. Comparison of the performance of the Zn-air battery prepared in this work and representative literature.

Air cathode	Catalyst loading ($mg\cdot cm^{-2}$)	Open circuit Voltage (V)	Specific capacity ($mAh\cdot g_{Zn}^{-1}$)	Voltage (V)	Reference
**D-Pd M**	**0.026**	**1.4**	**809**	**1.25 V @ 15 $mA\cdot cm^{-2}$**	**This work**
PdMo bimetallene	0.2	1.48	798	1.35 V @ 10 $mA\cdot cm^{-2}$	[14]
Pd−Gd2O3/C+RuO2	NA	1.46	724	1.3 V @ 5 $mA\cdot cm^{-2}$	[19]
Pd/B4C	0.6	1.43	NA	1.2 V @ 5 $mA\cdot cm^{-2}$	[20]
Commercial Pt/C + IrO_2	1	1.36	499	1.21@ 10$mA\cdot cm^{-2}$	[14]
Pd/CoOx/d-NC	1	1.476	760	1.2@ 10$mA\cdot cm^{-2}$	[21]
Ni SAs-Pd@NC	10	1.44	719.2	1.3 @ 5 $mA\cdot cm^{-2}$	[22]
$Pd_{55}Au_{45}$	2	1.44	821.4	1.3 V @ 10 $mA\cdot cm^{-2}$	[23]
PdMoCrW Tetrametallene	1	1.48	895	1.21@ 10$mA\cdot cm^{-2}$	[24]

S7 Reference

[1] W. Zhu, L. Zhang, P. Yang, C. Hu, Z. Luo, X. Chang, Z.-J. Zhao, J. Gong,] W Zhu,] L Zhang,] P Yang, C. Hu, Z. Luo, X. Chang, Z. Zhao, J. Gong, *Angewandte Chemie International Edition* **2018**, *57*, 11544–11548.

[2] Y. Li, Y. Yan, Y. Li, H. Zhang, D. Li, D. Yang, *CrystEngComm* **2015**, *17*, 1833–1838.

[3] G. Kresse, J. Furthmüller, *Comput Mater Sci* **1996**, *6*, 15–50.

[4] G. Kresse, J. Hafner, *Phys Rev B* **1994**, *49*, 14251.

[5] G. Kresse, D. Joubert, *Phys Rev B* **1999**, *59*, 1758.

[6] K. Lee, É. D. Murray, L. Kong, B. I. Lundqvist, D. C. Langreth, *Phys Rev B Condens Matter Mater Phys* **2010**, *82*, 081101.

[7] V. Wang, N. Xu, J. C. Liu, G. Tang, W. T. Geng, *Comput Phys Commun* **2021**, *267*, 108033.

[8] E. A. Nagul, I. D. McKelvie, P. Worsfold, S. D. Kolev, *Anal Chim Acta* **2015**, *890*, 60–82.

[9] H. Zhou, Z. Ma, G. Yang, X. Jiang, S. Duan, Y. Wu, M. Wang, L. Ni, L. Feng, G. Diao, *Batter Supercaps* **2024**, e202300563.

[10] L. Zhang, Z. Zhao, X. Fu, S. Zhu, Y. Min, Q. Xu, Q. Li, *ACS Appl Mater Interfaces* **2023**, *15*, 5198–5208.

[11] H. Yu, T. Zhou, Z. Wang, Y. Xu, X. Li, L. Wang, H. Wang, *Angewandte Chemie International Edition* **2021**, *60*, 12027–12031.

[12] Q. Yang, L. Shi, B. Yu, J. Xu, C. Wei, Y. Wang, H. Chen, *J Mater Chem A Mater* **2019**, *7*, 18846–18851.

[13] S. Huang, S. Lu, S. Gong, Q. Zhang, F. Duan, H. Zhu, H. Gu, W. Dong, M. Du, *ACS Nano* **2022**, *16*, 522–532.

[14] M. Luo, Z. Zhao, Y. Zhang, Y. Sun, Y. Xing, F. Lv, Y. Yang, X. Zhang, S. Hwang, Y. Qin, J. Y. Ma, F. Lin, D. Su, G. Lu, S. Guo, *Nature 2019 574:7776* **2019**, *574*, 81–85.

[15] C. Tang, N. Zhang, Y. Ji, Q. Shao, Y. Li, X. Xiao, X. Huang, *Nano Lett* **2019**, *19*, 1336–1342.

[16] L. Bu, C. Tang, Q. Shao, X. Zhu, X. Huang, *ACS Catal* **2018**, *8*, 4569–4575.

[17] Z. Wang, S. Xu, Q. Mao, K. Deng, Y. Xu, H. Wang, H. Yu, L. Wang, *Inorg Chem* **2023**, *62*, 13537–13543.

[18] H. Wang, Y. Li, S. Liu, H. Yu, K. Deng, Z. Wang, Y. Xu, L. Wang, *Chemical Communications* **2023**, *59*, 11101–11104.

[19] S. Ning, M. Li, X. Wang, D. Zhang, B. Zhang, C. Wang, D. Sun, Y. Tang, H. Li, K. Sun, G. Fu, *Angewandte Chemie International Edition* **2023**, *62*, e202314565.

[20] Y. N. Chen, X. Zhang, H. Cui, X. Zhang, Z. Xie, X. G. Wang, M. Jiao, Z. Zhou, *Energy Storage Mater* **2018**, *15*, 226–233.

[21] T. Kang, D. Nam, J. Kim, *Appl Surf Sci* **2022**, *582*, 152442.

[22] S. Wang, Z. Lin, M. Li, Z. Yu, M. Zhang, M. Gong, Y. Tang, X. Qiu, *J Mater Chem A Mater* **2022**, *10*, 6086–6095.

[23] S. Qiao, H. Shou, W. Xu, Y. Cao, Y. Zhou, Z. Wang, X. Wu, Q. He, L. Song, *Energy Environ Sci* **2023**, *16*, 5842–5851.

[24] Z. Zheng, K. Dong, X. Yang, Q. Yuan, *Langmuir* **2024**, DOI:10.1021/ACS.LANGMUIR.4C01196.

S8 Author contributions

Y.Z., R.L., S. Li, and C.S. conceived the project. R.L., S. Li, and C.S. supervised the project. Y.Z. carried out the material fabrication. Y.Z., F. F, D. G, and R.L. designed and performed electrocatalytic studies. R.L. Y.Z., and C.S. performed data analysis. S. Li. and Y. Z. performed XAS analyses. Z.C. and Y. Z. performed battery tests. K.C. and D.Z. performed (S)TEM analyses. N. M. and W. C. performed computational calculations. All authors co-wrote the manuscript.
